# Peptide dendrimer and hyaluronic acid modified nanovesicles for ocular delivery of timolol maleate and siRNA

**DOI:** 10.1038/s41598-025-10960-9

**Published:** 2025-07-18

**Authors:** Santoshi Naik, Naitik Jain, Nagarajan Theruveethi, Srinivas Mutalik

**Affiliations:** 1 Department of Pharmaceutics, Manipal College of Pharmaceutical Sciences, Manipal Academy of Higher Education, Manipal, Karnataka 576104 India; 2 Department of Optometry, Manipal College of Health Professions, Manipal Academy of Higher Education, Manipal, Karnataka 576104 India

**Keywords:** Glaucoma, Spanlastics, Nanovesicles, RNA interference, Peptide dendrimer, Targeted delivery, Materials science, Nanoscience and technology, Eye diseases

## Abstract

Glaucoma, a leading cause of irreversible blindness, is characterized by retinal ganglion cell (RGC) degeneration due to elevated intraocular pressure (IOP) and apoptosis. While timolol maleate effectively lowers IOP, it does not prevent RGC loss and suffers from poor corneal permeability and rapid clearance. This study introduces a novel dual-delivery nanovesicular system employing multifunctional spanlastics to simultaneously lower IOP and inhibit RGC apoptosis via caspase-2 gene silencing. The system comprises two distinct nanovesicle populations: (i) timolol-loaded vesicles conjugated with peptide dendrimers to enhance corneal penetration and anterior segment delivery; and (ii) siRNA-loaded vesicles targeting Caspase-2, coated with hyaluronic acid for posterior segment delivery and gene silencing. This is the first approach integrating IOP reduction with targeted genetic intervention to protect RGCs. Formulations were optimized using a Design of Experiments approach and showed desirable physicochemical properties, sustained release, improved transcorneal permeability, and 1-month stability at 4 °C. In vitro studies confirmed Caspase-2 silencing and apoptosis reduction in RGC-5 cells, while in vivo results demonstrated prolonged IOP control. Safety was confirmed via histopathological and ocular irritation assessments. This targeted, non-invasive dual-delivery platform offers a promising therapeutic strategy for comprehensive glaucoma management.

**Supplementary Information:**

The online version contains supplementary material available at 10.1038/s41598-025-10960-9.

## Introduction

Glaucoma is a progressive neurodegenerative disease marked by elevated intraocular pressure (IOP) and gradual loss of retinal ganglion cells (RGCs) and their axons, ultimately leading to irreversible blindness^1^. In 2022, the global incidence of primary open-angle glaucoma (POAG) among individuals aged 40–79 was estimated at 23.46 cases per 10,000 person-years^2^. The burden of glaucoma is expected to rise significantly, with projections indicating 11.8 million cases by 2040. Notably, POAG is more prevalent in men than women, as suggested by a Bayesian meta-regression analysis^3^.

Currently, the management of glaucoma primarily focuses on the reduction of elevated IOP using various agents like prostaglandin analogs, β-blockers, carbonic anhydrase inhibitors, adrenergic agonists, parasympathomimetics, and Rho-kinase inhibitors^4^. Among all, Timolol Maleate (TM), a non-selective β-blocker, remains the gold standard even after 47 years since its introduction in 1978. Despite its proven efficacy, the clinical benefits of TM are limited by challenges such as poor ocular bioavailability, low corneal permeability, and the need for frequent administration, all of which result in suboptimal therapeutic outcomes and poor patient compliance^5^. Additionally, 80% of a topically applied timolol eye drop drains through the nasolacrimal duct and is systemically absorbed, with absorbed timolol potentially causing severe cardiac and respiratory side effects^6^.

To address these limitations, various nanocarrier-based delivery platforms, namely solid lipid nanoparticles, niosomes, polymeric nanoparticles, and liposomes have been explored to enhance TM bioavailability and prolong drug residence time^7–9^. Although these approaches have improved corneal penetration and provide sustained release, they still face problems like poor targeting and the risk of causing eye irritation or side effects. In parallel, there is increasing recognition that elevated IOP alone does not fully explain glaucoma progression. RGC apoptosis and axonal degeneration at the lamina cribrosa are key contributors to the neurodegeneration observed in glaucoma^10^. While current therapies effectively lower IOP, they fail to address these underlying neurodegenerative mechanisms^11^. Caspase-2, an initiator of neuronal apoptosis, has emerged as a promising neuroprotective target. Inhibition of *caspase-2* has shown potential to preserve RGC integrity and function^12^. siRNA-based therapies like QPI-1007, designed to silence caspase-2^13^, showed early clinical potential but did not meet efficacy endpoints in larger trials (NCT02341560), likely due to poor tissue targeting, limited cellular uptake, and siRNA instability within ocular environments.

**Fig. 1:**
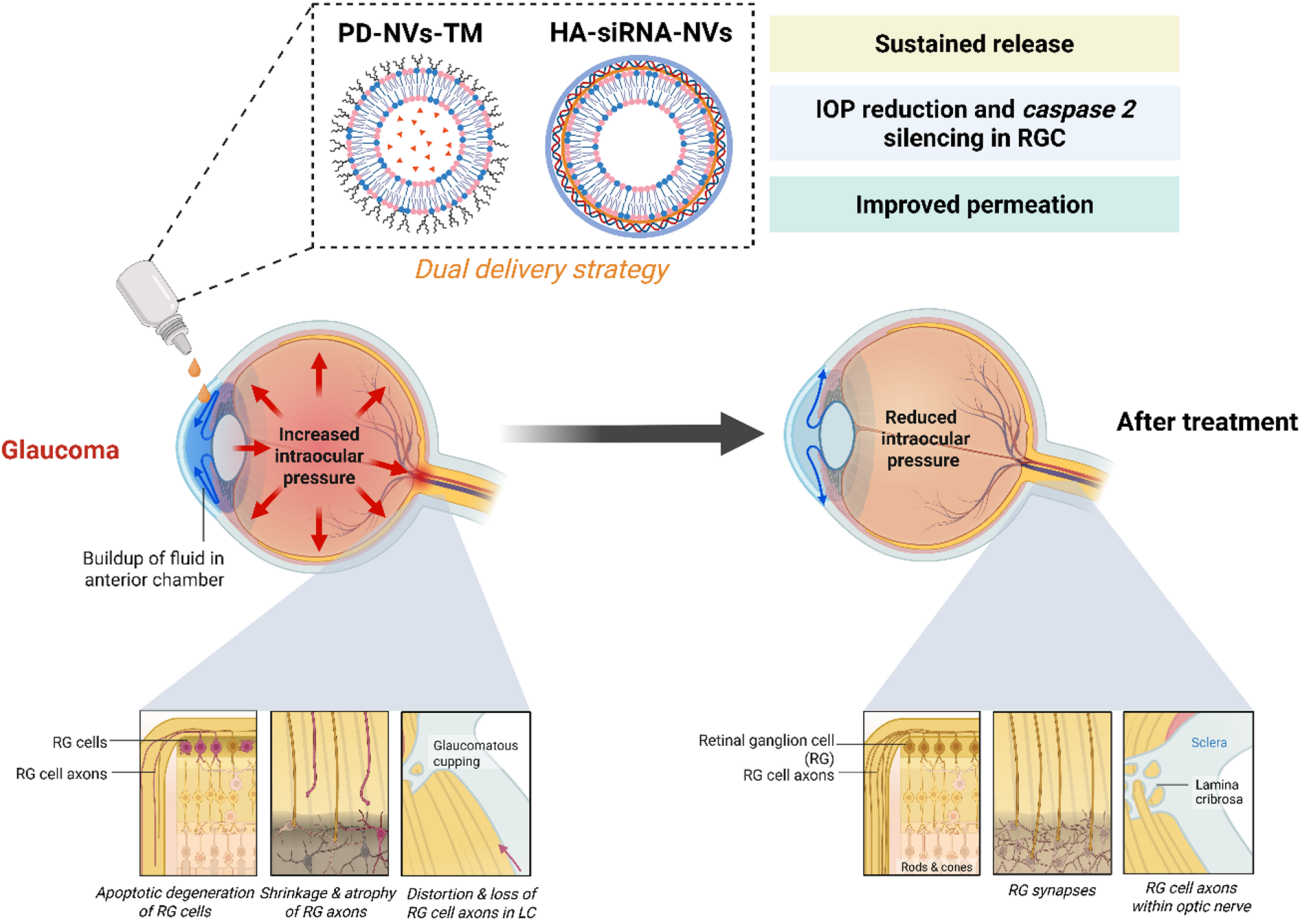
Schematic illustration of the proposed dual delivery strategy for glaucoma treatment. Created with BioRender.com.

To address the limitations of conventional glaucoma treatments, this study presents a dual-delivery strategy using spanlastics, elastic nanovesicles formulated separately with Maleate TM and Caspase-2 siRNA. Though both therapeutics are delivered via spanlastics, each is encapsulated in a distinct formulation to preserve stability and functional integrity. TM-loaded vesicles were conjugated with peptide dendrimers (PD) to enhance corneal permeability, while siRNA-loaded vesicles were functionalized with hyaluronic acid to achieve targeted delivery to RGCs (Fig. 1). This platform aims to lower IOP and simultaneously silencing the *caspase-2* gene implicated in RGC degeneration. By integrating these complementary mechanisms into a minimally invasive, biocompatible system, the approach offers the potential for improved therapeutic efficacy and long-term glaucoma management.

## Materials and methodology

### Materials

Timolol maleate was kindly supplied as a gift sample by Orbicular Pharmaceutical Technologies (Hyderabad, India). DSPE-PEG2000-COOH was sourced from Nanosoft Biotechnology (USA). Polyethylene imine (Mw ~ 25,000), Hyaluronic acid sodium salt, Fluorescein-5-isothiocyanate (*FITC*), SYBR Green, 1-Ethyl-3-(3-dimethyl aminopropyl) carbodiimide (EDC), 2-(N-morpholino) ethanesulfonic acid, N-Hydroxysuccinimide (NHS) and mannitol were procured from Sigma-Aldrich (USA). RNA primers were procured from Eurofins Scientific (India) and the sequences are listed as follows: mouse caspase-2 (Forward: 5′-TCATCCAAGCATGTCGTGGAGG-3′, reverse 5′-GCAGTGAACAGAAGGAGGTGCC-3′), mouse beta-actin: (forward 5′-GATCATTGCTCCTCCTGAGC -3′, reverse 5′-AGTCCGCCTAGAAGCACTTG -3′). Reagents and solvents used in the study were of analytical or HPLC grade. The human corneal epithelial cells (HCE cells) were procured from the ATCC (USA). DMEM was sourced from Gibco-Thermo Fisher Scientific, and D-PBS along with FBS were procured from Himedia Laboratories, both located in Mumbai, India.

### Methods

#### Synthesis, purification and characterization of Peptide Dendrimers

Arginine (Arg)- terminated PD having 8+ charge was constructed by Fmoc based solid phase peptide synthesis (SPSS) from C to N-terminal of Glycine-Leucine-Lysine-(Lysine-(Arginine)2)2 following our previously reported method^14^. Briefly, chlorotrityl chloride resin was first swollen in DMF and activated with 20% (v/v) piperidine in DMF. Fmoc-Gly-OH coupling was performed post-washing with DMF using HBTU and DIEA, Subsequently, exposed to 20% (v/v) piperidine solution in DMF. Subsequent amino acids were sequentially coupled after confirming ≥ 99% efficiency via the Ninhydrin test. This cycle of deprotection and amino acid coupling was repeated until the required PD chain length was achieved. The synthesized PD was cleaved from the resin using piperidine in DMF. The dried resin was stirred, and the residue was subjected to azeotropic distillation with toluene and then triturated with ice-cold diethyl ether. The final product was dissolved in deionized water, freeze-dried and kept at 2–8 °C until use.

The constructed PD was purified using RP-HPLC (Shimadzu, Kyoto, Japan) using a Kromasil RP C18 column (5 µm, 250 × 4.6 mm) and was characterized by ESI+-MS (QTRAP LC/MS/MS system) for determining molecular weight, ^1^HNMR analysis to assess the chemical shifts of protons and RP-HPLC for single peak purity. The PDs were further analyzed using FT-IR (Shimadzu, Kyoto, Japan) to ascertain the presence of key functional groups, and by thermal analysis (DSC-60, Shimadzu, Kyoto, Japan) to assess the melting behavior of the incorporated amino acids.

#### Design of target siRNA

siRNAs targeting the caspase 2 gene (GenBank: NM_007610.2) were designed using various bioinformatics tools, including siPRED (Elixir biotools), siDirect (University of Tokyo), siRNA Target Finder (GeneScript, Piscataway, NJ, USA), Block-iT RNAi Designer (Invitrogen), and Eurofins Genomics (Eurofins Scientific Group). Selection criteria included > 80% inhibition efficiency, GC content of 30–52% (as per Reynold et al.), and minimal self-complementarity. The designed siRNAs, targeting different caspase 2 mRNA regions (Supplementary Information) were purchased from Sigma-Aldrich.

**Table 1:** Results of stability studies of optimized nanovesicles.

Storage condition	Days	Particle size (nm)	PDI	EE%	Aggregation
Room temperature (25 ± 2 °C)	0 day	178.9 ± 4.21	0.202 ± 0.018	100.00 ± 0.00	−
	15 days	212.8 ± 3.39	0.316 ± 0.009	72.17 ± 1.77	+
	30 days	260.9 ± 3.96	0.342 ± 0.023	65.01 ± 1.24	+
Refrigerated temperature (4 ± 2 °C)	0 day	178.9 ± 4.21	0.202 ± 0.018	100.00 ± 0.00	−
	15 days	179.9 ± 4.66	0.218 ± 0.003	93.72 ± 0.89	−
	30 days	182.0 ± 1.37	0.240 ± 0.121	80.11 ± 1.45	−

−: no aggregation, +: mild aggregation.

#### Primer design and validation

Primers used for gene expression analysis were custom-designed and procured from Eurofins (Bangalore, India). Gradient PCR was carried out with 50 ng of constructed cDNA to identify the optimal annealing temperature. The *Caspase-2* gene-specific primer and Beta-actin gene primer was validated through PCR using a mixed cDNA pool from the target cells. Further validation was conducted using SYBR-based amplification and melt curve analysis.

#### Formulation of TM-loaded nanovesicles

Drug-excipient compatibility was evaluated using FTIR spectroscopy and DSC analysis to assess interactions between TM and the formulation excipients of elastic nanovesicles. The results are discussed in Supplementary Information and shown in Figure S1 (Supplementary Information). TM-loaded nanovesicles (TM-NVs) were prepared according to a previously published ethanol injection technique with slight modifications. Briefly, TM and Span 60 were solubilized in ethanol, sonicated, and then injected at a steady rate of 1 mL/min into a preheated (70 °C) aqueous phase comprising Tween 80. The dispersion was stirred at 70 °C for 30 min using a magnetic Stirrer with temperature control, then cooled to room temperature, stirred for another 30 min, and bath-sonicated for 4 min to prevent vesicle aggregation.

#### Optimization and validation of TM-loaded nanovesicles

Box-Behnken design (BBD) was employed to assess the influence of independent variables such as Span 60 concentration (A), Stirring speed (B), and sonication time (C) on responses, namely, particle size (R1) and entrapment efficiency (R2)^15^ and optimize the composition of nanovesicles using Design Expert® software (v.10.0.3.1; Stat-Ease, Inc., Minneapolis, USA). Table S1 in Supplementary Information summarizes the study design.

The responses were optimized with specific targets, for example, a minimum for particle size and a maximum for entrapment efficiency (EE). The design space indicated 15 experimental runs (Table S2; Supplementary Information), each varying across two measurable parameter across three defined concentration levels. These runs were intended to assess the effects of individual factors, interactions between factors, and quadratic effects of the chosen variables^16^. ANOVA assessed statistical significance, while R^2^ and Q^2^ values validated model reliability and predictive accuracy. The confirmation tool compared predicted and experimental responses, ensuring validity if the results fell within the prediction interval. The most desirable prediction was experimentally tested, with accuracy evaluated using percent relative error.

$$\text{Percent relative error}=\frac{\text{Predicted response}-\text{Observed response}}{\text{Predicted response}}\times 100$$

#### Preparation of PD-conjugated nanovesicles

The peptide dendrimer was covalently conjugated to carboxyl-functionalized nanovesicles (NVs-DSPE-PEG-COOH2000) using EDC-NHS chemistry. Briefly, an excess amount of EDC was added to DSPE-PEG and agitated for 45 min under ambient conditions, followed by the addition of NHS and further stirred for 12 h. Unreacted EDC and NHS were removed via dialysis, after which excess PD was introduced and stirred for 24 h to achieve complete conjugation. The formulation was preserved with 0.01% benzalkonium chloride and filtered through sterile nylon filters (0.22 μm). Finally, excess PD was removed by dialysis, and the conjugated nanovesicle dispersion (PD-NVs-TM) was lyophilized using 2.5% cryoprotectant.

#### Preparation of HA-coated siRNA-loaded nanovesicles

A layer-by-layer approach was employed to prepare HA-coated siRNA-loaded nanovesicles (HA-siRNA-NVs). First, nanovesicles were formulated via the ethanol injection and stabilized with 1 mg/mL Polyethyleneimine (PEI, MW 25 kDa). The PEI solution was added to the nanovesicle dispersion was stirred for 30 min, subsequently spun at 10,000 rpm for 10 min to eliminate any excess PEI. The PEI-coated nanovesicles were lyophilized with 2% mannitol as a cryoprotectant, followed by size, zeta potential (ZP), and polydispersity index (PDI) analysis. The lyophilized PEI-coated nanovesicles (1 mg/mL) were resuspended in water and added dropwise to a stirred solution of siRNA (4.22 µg/µL) and cationized hyaluronic acid (HA, 1 mg/mL) under aseptic conditions. The mixture was agitated for 1 h, held at 37 °C for 0.5 h, and filtered. Unbound polyelectrolytes were separated via centrifugation at 3000 rpm for 10 min, and the formulation was lyophilized with 2% mannitol. The siRNA adsorption onto the PEI-coated nanovesicles was quantified by measuring the siRNA concentration in the supernatant using the Epoch 2 microplate reader at 260/280 nm. The average of four readings was multiplied by the dilution factor. Average particle size, ZP, and PDI of the final siRNA-loaded nanovesicles were measured using the Zeta Sizer (Zetasizer Nano ZS, Malvern, UK).

#### Characterization of unconjugated and PD-conjugated nanovesicles

The average particle size, PDI, and ZP of nanovesicles were measured using a Zetasizer. PD-conjugated nanovesicles were analyzed by FTIR and ^1^H NMR to confirm amide bond formation between PD’s terminal amine groups and the carboxyl group of DSPE-PEG-COOH2000 in NVs-TM. FTIR (FTIR-8300, Shimadzu, Japan) was performed by dispersing the drug in KBr, compressing it into discs at 5-ton pressure, and recording spectra (4000–400 cm⁻1). Proton (^1^H) NMR spectroscopy was performed using a 400 MHz solid-state NMR spectrometer to confirm the conjugation of PD to NVs-TM. The sample was dissolved in deuterated chloroform before analysis. DSC was used to analyze the thermal behaviour of pure TM, mannitol, NVs-TM, PD-NVs-TM using a DSC-60 (Shimadzu Corporation, Kyoto, Japan). The thermal analysis curves were recorded by tracking heat flow with respect to temperature. Samples were heated from 30 °C to 350 °C. An X-ray diffractometer (Rigaku Ultima IV, Japan) was used to analyze the XRD patterns of pure TM, NVs-TM, and PD-NVs-TM. The morphology and surface characteristics of nanovesicles were determined using TEM (FEI Tecnai T12, Netherlands). A drop of the sample was dispensed to a carbon film-coated copper grid and air-dried for 2 min. The sample was then stained with 2% w/v phosphotungstic acid, followed by drying. Analysis of the sample-loaded copper grid was carried out at 200 kV.

The nanovesicle dispersion was spun at 20,000 rpm at 4 °C for 0.5 h. The collected was resuspended in hydration media with 5% w/v mannitol as a cryoprotectant. The dispersion was then frozen at − 80 °C for 12 h and freeze-dried at − 55 °C for 48 h using a lyophillizer (Alpha 1–2 LD Plus).

#### Determination of elasticity

The deformability index (DI) was employed to express the properties of elastic nanovesicles^17^. It was assessed by extruding the vesicles through a filter (pore size of 200 nm; diameter 25 mm) at a steady flow rate of 1 mL/min, regulated by the syringe pump (Harvard Apparatus Pump, USA), and size alteration was determined. The elasticity was assessed based on the time required for extruding elastic nanovesicles through the membrane. The study was performed in triplicate, and DI was determined using the formula given below:

$${\text{DI}} = {\text{j}}/{\text{t}}({\text{R}}_{{\text{v}}} /{\text{R}}_{{\text{p}}} )^{2} \times 100$$

where j denotes the volume of extruded nanovesicles (mL), t is the extrusion time (s), Rv indicates the size of the vesicles post-extrusion (nm), and Rp refers to the pore size of the barrier (nm).

#### Determination of in vitro drug release

The release of TM from NVs-TM and PD-NVs-TM was evaluated using a dialysis bag diffusion method^18^. Briefly, 0.5 mL of the TM-loaded nanovesicle formulation was placed inside a dialysis bag (MWCO 12,000 Da), which was then suspended in 50 ml PBS (pH 7.4) maintained at 37 ± 1 °C in a shaker set at 90 rpm. At set timepoints (0.5, 1, 2, 4, 6, 8, 10, 12, and 24 h), 1 mL of aliquot was withdrawn and replaced with PBS (pH 7.4) to maintain sink conditions. The study was conducted in triplicate.

#### Ex vivo Corneal permeation studies

To evaluate the permeation of the drug and its formulations, a vertical diffusion cell (0.78 cm^2^) was utilized^19^. Freshly harvested corneas were carefully positioned between the donor and receptor compartments, with the endothelial side facing the donor chamber and the epithelial side facing the receptor chamber. 5 mL of pH 7.4 GBR buffer was added to the receptor compartment, maintained at 37 ± 0.5 °C, and agitated continuously at 50 rpm using a magnetic stirrer to simulate blinking. 1 mL aliquot of each formulation, free TM, MF, NVs-TM, and PD-NVs-TM, was introduced in the donor chamber. At specific time points (0.5, 1, 2, 3, 4, 6, 8, 10, 12, 24, and 48 h), 0.5 mL aliquots were withdrawn from the receptor compartment and replaced with fresh GBR buffer (pH 7.4) to ensure sink conditions. The study was performed in triplicate. The amount of TM penetrated the cornea was quantified using HPLC analysis at 295 nm, based on a method we developed and published previously^20^.

#### Corneal hydration level

Post-permeation studies, the corneal hydration level was assessed to evaluate the integrity of corneal tissues, following a previously reported method^21^. Briefly, the weight of each cornea (W_a_) exposed to the GBR buffer was recorded, then dried at 60 °C in an oven and reweighed (W_β_). The hydration level was assessed using the formula as shown below:

$${\text{Hydration}}\,{\text{Level}}\,(\% ) = 1 - \frac{{{\text{W}}2}}{{{\text{W}}1}} \times 100$$

#### Stability study

The stability of the optimized elastic nanovesicles was assessed by evaluating drug leaching during storage. The formulation was stored in sealed amber-colored glass ampoules at 25 ± 2 °C and 4 ± 2 °C for 1 month. Aliquots were collected at 0, 15, and 30 days and analyzed for particle size, PDI, and %EE^22^. Additionally, microscopic examination was performed to assess particle aggregation over time.

#### In vitro cell culture studies

##### Cell viability assay

The MTT assay evaluated cell viability in HCE-2 and RGC-5 cell lines. HCE-2 cells were treated with TM solution, NVs-TM, and PD-NVs-TM, while RGC-5 cells received TM solution, HA-NVs, and HA-siRNA-NVs at concentrations of 0.187–6.25 mg/mL. Post 24 h of incubation at 37 °C in a 5% CO_2_ atmosphere, MTT reagent was introduced and allowed to incubate for an additional 4 h. The obtained formazan crystals were solubilized in DMSO, and the absorbance was recorded at 570 nm using a plate reader (BioTek Instruments Inc., US). % Cell viability was then calculated.

$$\text {viability} =\frac {\text {Absorbance of treated cells}} {\text {Absorbance of untreated cells}} \times 100$$

##### Cellular uptake studies

To access the cellular internalization of prepared nanovesicles, RGC-5 cells were plated in a 6-well plate at 2 × 10^5^ cells/well density and were incubated for 24 h at 37 °C with CO_2_. Later, the cells were treated with 10 µg/mL FITC-labeled unconjugated and conjugated nanovesicles or 1.5 µg/mL FITC solution as a control. Following 24 h incubation, cells were rinsed thrice with DPBS and trypsinised and cells were re-dispersed in DPBS and placed in FACS tube for analysis using flow cytometry (BD CellQuest Pro v6.0).

##### siRNA transfection

siRNAs and Lipofectamine 2000 were separately diluted in siRNA transfection medium and reduced-serum medium, respectively. The diluted solutions were then combined and held at ambient temperature for 15–30 min to allow complex formation. The prepared complexes were added to each well containing cells and transfection medium, while control wells received only the transfection reagent. Cells were incubated at CO_2_ incubator for 6 h, after which DMEM supplemented with 20% FBS was added to support cell viability. The cells were maintained under the same conditions, and siRNA-mediated *caspase-2* gene silencing was evaluated 48 h post-transfection in 6-well plates using quantitative real-time PCR (qRT-PCR) as discussed below:

Post-treatment, the culture medium was collected from all wells into polystyrene tubes. After discarding the PBS, 350 μL of trypsin–EDTA solution was added, and cells were incubated at 37 °C for 3–4 min to facilitate detachment. The detached cells were resuspended by reintroducing the culture medium into the wells and subsequently transferred to polystyrene tubes for collection. The cell suspension was centrifuged, and the supernatant was carefully discarded. The resulting cell pellet was rinsed twice with PBS to ensure the complete removal of residual buffer. Total RNA was extracted with Qiagen RNeasy kit, and DNase treatment was subsequently performed to eliminate genomic DNA contamination, yielding high-purity RNA suitable for downstream applications.

##### In vivo safety and efficacy

All experimental procedures and animal care were conducted in compliance with the CCSEA (Committee for Control and Supervision of Experiments on Animals, Ministry of Fisheries, Animal Husbandry and Dairying, Government of India) guidelines and adhered to the ARRIVE guidelines. The animal study protocols were reviewed and approved by the Institutional Animal Ethics Committee of Kasturba Medical College, MAHE, Manipal (Approval No: IAEC/KMC/120/2020). Efforts were made to use the fewest number of rats necessary, ensuring ethical and responsible use.

##### Pharmacodynamics study

The therapeutic effectiveness of the optimized nanovesicles in reducing IOP was assessed in male Sprague–Dawley rats (200–250 g) and compared with commercially available eye drops. The rats were segregated into five groups (n = 3 each): Group I: Untreated control; Group II: Normal saline-treated control; Group III: Marketed formulation (0.5% w/v Timolol maleate); Group IV: NVs-TM; Group V: PD-NVs-TM. Glaucoma was induced by injecting 50 µL of 10% NaCl into the episcleral vein, and IOP was reassessed after 30 min. The right eye received treatment, while the left eye served as a control. IOP was measured using an iCare Home Tonometer, and formulations were administered accordingly. IOP was monitored daily for 1 week. Intraperitoneal administration of xylazine (13 mg/kg) and ketamine (100 mg/kg) was used to anesthetize SD rats. Upon completion of the study, rats were euthanized, and IOP data were recorded using iCare LINK software (Topcon Beijing Ltd., Hong Kong).

##### Ocular irritation potential

The ocular safety of the developed formulations was assessed in Sprague–Dawley (SD) rats following OECD Guideline 405. The study included three groups (n = 3 each): Group I: TM solution (0.5% w/v); Group II: NVs-TM; Group III: PD-NVs-TM. Prior to testing, a slit lamp biomicroscope (Keeler Ltd. SL4 4AA, UK) was used to confirm the absence of pre-existing ocular irritation or corneal damage, and digital images were recorded for reference. To minimize discomfort, 1–2 drops of topical anesthetic (0.5% proparacaine HCl) were instilled before administering 0.1 mL of the test material into the right eye, while the left eye served as a control. For acute eye irritation, macroscopic evaluations were performed at 1, 24, 48, and 72 h post-application using a slit lamp biomicroscope. Fluorescein sodium (1 mg) was used to detect corneal epithelial damage at 1, 24, and 48 h, while Lissamine Green (1.5 mg) was applied at 72 h to assess cell degeneration. Ocular lesions were graded according to the Draize scoring rules^23^. For long-term safety evaluation, the test formulations (0.1 mL) were administered five times daily at 5 min intervals for 1 week. After the final dose, the same slit lamp biomicroscopy and staining procedures were performed at 1, 24, 48, and 72 h to monitor cumulative ocular effect.

##### Histopathological analysis

Following the chronic eye irritation study, the enucleated eye bulbs were immediately fixed in 10% formalin for 48 h. Subsequently, the iris, cornea, retina, and ciliary body were dissected and processed through a graded series of alcohol and xylene before being embedded in paraffin wax. Thin tissue sections were obtained using a microtome, floated in a 50–52 °C water bath, and mounted onto microscopic slides. The sections were then dyed with hematoxylin and eosin (H&E) and visualized under a Trinocular microscope (LX-500 LED, Labomed Inc., USA) to assess histopathological alterations. High-resolution images were clicked using a MiaCam CMOS AR 6 pro microscope camera (Labomed Inc., USA) and analyzed with Picture AR Pro software (Fahmy et al., 2021; Huang et al., 2017).

#### Statistical analysis

The data are shown as means ± standard deviations. Statistical significance between groups was determined using either one-way or two-way analysis of variance (ANOVA) followed by Tukey’s post hoc test. All statistical analyses were conducted with GraphPad Prism 8 (GraphPad Software, San Diego, CA, USA).

## Results and discussion

### Synthesis, purification and characterization of Peptide Dendrimer

Arginine-terminated PD with an 8 + charge was constructed using the Fmoc-solid phase peptide synthesis (Figure S2; Supplementary Information). Due to multiple arginine and lysine residues, the peptide dendrimer exhibited strong cationic properties. Arginine, with its guanidine groups (pKa ~ 12.5), remained protonated under physiological conditions (pH ~ 7.4), while lysine contributed additional positive charges through its primary amine groups^24^. The synthesized PD had a MW of 1197.5 g/mol, a 75% yield, and the sequence Gly-Leu-Lys-(Lys-(Arg)_2_)_2_ from C- to N-terminus. HPLC analysis confirmed its purity (> 95%) with a single peak with a retention time of 9.450 min (Figure S3 D; Supplementary Information). This study explored the potential of a custom-synthesized PD as a permeation enhancer for ocular drug delivery. It has been reported that PDs enhance the cellular uptake of nanocarriers through electrostatic interactions between the positively charged arginine residues and the negatively charged cell membrane^25^. Additionally, lysine residues contribute to this enhanced permeability by further destabilizing the cell membrane and facilitating nanocarrier entry^26^. The combined effect of arginine and lysine within the PD structure is likely responsible for the improved transcorneal permeation observed in this study.

Synthesized PD was characterized using ESI–MS, FTIR, DSC, and NMR to assess its structural and physicochemical properties. The ESI–MS spectrum exhibited peaks corresponding to (M + 2H)^2^⁺ and (M + 3H)^3^⁺ species, while (M + H⁺) peak was not observed. This charge distribution aligns with the expected ionization behavior of large peptides. The molecular weight was determined by deconvoluting the charge states using the equation M = z × (m/z) − zH. Based on the detected m/z values of approximately 600 for (M + 2H)^2^⁺ and 400 for (M + 3H)^3^⁺, the calculated molecular weight of PD was 1198 Da, which closely aligns with the theoretical value of 1197.5 Da. These findings confirm the successful synthesis of PD (Figure S3 E; Supplementary Information). The proton NMR spectrum of PD exhibited a doublet peak at 0.8 ppm, corresponding to the hydrogen atoms attached to isopropyl group in leucine. Chemical shifts at 2.5 ppm and 3.1–3.6 ppm were attributed to the methylene (-CH_2_-NH) and methine (-CH_2_-NH-CH = NH) protons of arginine respectively (Figure S3 C; Supplementary Information). Additionally, shifts between 4.21 and 4.32 ppm corresponded to methine protons adjacent to NH and -NH_2_ groups^27^. The DSC profile of PD (Figure S3 B; Supplementary Information) displayed endothermic peaks within the 200–230 °C range, which align with the melting points of its amino acid components: lysine (215 °C), arginine (222 °C), and glycine (233 °C)^28^. The FTIR analysis of PD (Figure S3 A; Supplementary Information) displayed a distinct peak at 1659.53 cm^1^, corresponding to the carbonyl (–C=O) functional group. A peak at 1538.50 cm^−1^ was attributed to the hydroxyl group present in arginine. Additionally, a broad absorption band at 3275.96 cm^−1^ was associated with the aliphatic (-CH_2_) group of lysine^29^.

### siRNA design and primer design & validation

Based on performance in multiple siRNA design tools (siPRED, siDirect, Block-iT, GeneScript, and Eurofins Genomics), siRNA-6 was selected from six candidates (siRNA-1 to siRNA-6, as shown in Table S3; Supplementary Information. It met all key criteria, including 82.1% inhibition efficiency, 47% GC content, and no self-complementarity. The siRNAs comprise 19 nucleotides with two 3’ thymidine (T) overhangs. All sequences were blasted against the mouse genome to minimize off-target effects. A scrambled siRNA was used as a negative control.

The optimum annealing temperature for all primers was determined to be 55 °C to ensure specificity and efficiency. Caspase-2 and beta-actin gene-specific primers were validated by PCR using a mixed cDNA pool from the cells. Validation was further confirmed through SYBR amplification (Figure S4 A; Supplementary Information) and melt curve analysis (Figures S4 B-C; Supplementary Information). All the primers produced the expected product size without self-annealing or dimerization.

### Preliminary screening of excipients

Preliminary studies revealed that formulations containing Tween 80 demonstrated significantly higher entrapment efficiency in comparison to Tween 40 and Tween 20. This can be attributed to the HLB values of the edge activators, 15.0 for Tween 80, 15.6 for Tween 40, and 16.7 for Tween 20, where a lower HLB correlates with increased hydrophobicity and enhanced drug entrapment. The longer alkyl chains in Tween 80 contribute to reduced membrane fluidity and fewer hydrophilic pores, promoting greater encapsulation^30^. Additionally, Tween 80 exhibited the highest deformability due to its flexible, non-bulky hydrocarbon chains^31^. Among the non-ionic surfactants tested, Span 60 produced the most stable vesicles, outperforming Span 40 and Span 80. The long alkyl chain in Span 60 enhances membrane rigidity and vesicle stability, while Span 80 leads to vesicle disruption and aggregation, and Span 40 showed moderate stability but was less effective than Span 60 in this study. The drug-excipient compatibility studies are described in the supplementary file.

### Formulation of TM-loaded nanovesicles

The TM loaded nanovesicles were formulated by solvent injection technique using span 60, tween 80 and TM. The synthesized peptide dendrimer were incorporated into the optimized nanovesicles by EDC-NHS chemistry.

### Optimization of nanovesicles

Quality by Design approach was employed using a BBD to optimize the nanovesicular formulation by evaluating the effects of Span 60 concentration (A), stirring speed (B), and bath sonication time (C). The outcomes measured were particle size (R1) and entrapment efficiency (R2), with 15 experimental runs performed as shown in Table S4 (Supplementary Information).

#### Influence of formulation variables on particle size and entrapment efficiency

Particle size ranged from 173.6 to 495.6 nm, while entrapment efficiency varied between 36.19 and 96.03%, underscoring the considerable influence of the selected formulation variables (Table S5; Supplementary Information). Statistical analysis affirmed the robustness and significance of the developed models, with p-values of 0.0105 for particle size and 0.0187 for entrapment efficiency (Table S6; Supplementary Information). The models exhibited strong predictive capabilities, as reflected in the F-values (6.126 for particle size and 5.107 for entrapment efficiency) with high regression coefficient values of 0.9994 and 0.9928, respectively.

Among the independent variables, Span 60 concentration (*p* < 0.05) had the most significant impact on both responses. An increase in Span 60 concentration led to larger particle sizes and enhanced entrapment efficiency, likely due to increased lipid content promoting vesicle growth and improved drug encapsulation. In contrast, stirring speed exerted a negative influence—decreasing particle size (coefficient = − 34.08) and entrapment efficiency (coefficient = − 0.51)—possibly due to intensified shear forces that disrupt vesicular structures and promote drug leakage. Bath sonication time demonstrated a minimal and statistically non-significant effect (*p* > 0.05), though extended sonication showed a slight reduction in both responses. These trends were visually supported by the contour and 3D surface plots (Figure S5; Supplementary Information), which illustrated the interactions between formulation variables and responses. A clear positive correlation was observed between Span 60 concentration and both particle size and entrapment efficiency, while increased stirring speed consistently reduced both parameters—aligning with the hypothesis that greater mechanical energy compromises vesicle integrity. These graphical trends corroborate the statistical results and provide further mechanistic insight into the formulation behaviour.

#### Optimization and model validation

The objective of the optimization was to minimize particle size while maximizing entrapment efficiency, within the predefined ranges of the independent variables (Table S5; Supplementary Information). Using Design-Expert® software, the optimal formulation was identified as comprising 70.30 mg of Span 60, a stirring speed of 1147.41 rpm, and a bath sonication for 3.32 min. This combination was predicted to yield a particle size of 170.20 nm and an entrapment efficiency of 60.68%, achieving a desirability score of 1.0, indicative of an ideal balance between both responses. To validate the model, the optimized formulation was prepared experimentally. The resulting particle size was 178.9 ± 7.3 nm, and the entrapment efficiency was 58.68 ± 6.5%. The percent relative error of 5.11% for particle size and + 3.24% for entrapment efficiency was within the acceptable threshold of ± 15%, confirming the accuracy, reliability, and robustness of the predictive model.

### Preparation of PD-conjugated nanovesicles by EDC-NHS chemistry

The PD-NVs-TM were successfully obtained, as confirmed by FTIR, DSC, and NMR analyses, indicating stable amide bond formation and successful surface modification. Post-conjugation, the nanovesicles maintained structural integrity with a slight increase in particle size and zeta potential, suggesting successful surface conjugation without compromising vesicle stability. The PDI remained within acceptable limits, reflecting uniform particle distribution, while %EE) was only marginally affected. These findings confirm the successful conjugation of the peptide dendrimer and its minimal impact on the physicochemical characteristics of the nanovesicles.

### Preparation of HA-coated siRNA-loaded nanovesicles by layer-by-layer approach

**Fig. 2:**
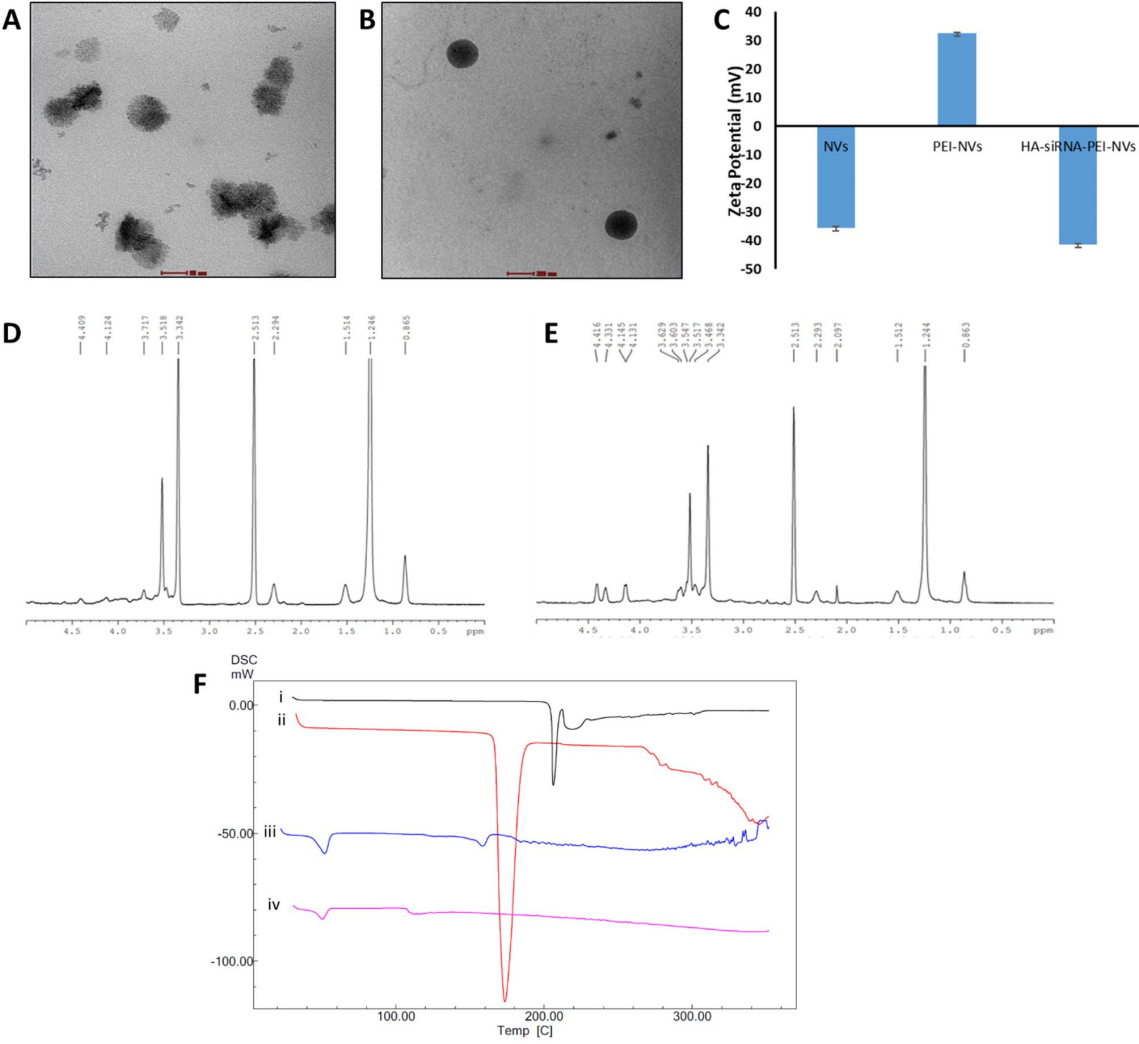
TEM imaging of nanovesicles (**A**) blank nanovesicles (Scale: 50 nm); (**B**) PD-conjugated nanovesicles (Scale: 200 nm); (**C**) Zeta potential analysis of nanovesicles to confirm polyelectrolyte coating; ^1^HNMR spectra of nanovesicles (**D**) and PD-NVs-TM (**E**); (**F**) DSC curves of (i) Pure TM, (ii) Mannitol, (iii) NVs-TM and (iv) PD-NVs-TM.

The successful surface modification of nanovesicles with PEI was confirmed through zeta potential measurements. Uncoated nanovesicles exhibited a negative ZP of − 35.5 mV, which reversed to + 32.5 mV following PEI coating, indicating the effective adsorption of the strongly cationic polymer. Subsequent layer-by-layer coating with polyelectrolytes (siRNA and HA) resulted in a marked shift to a highly negative ZP of − 41.3 mV, confirming the successful deposition of HA on the vesicle surface (Fig. 2C). The molar concentration of siRNA was determined to be 317.5 µM (equivalent to 4.22 µg/µL). These surface charge transitions validate the sequential layering of PEI, siRNA, and HA. Table S7 (Supplementary Information) summarizes the physicochemical characteristics—particle size, PDI, ZP, and %EE of both blank and surface-modified nanovesicles. While particles below 200 nm favor passive corneal transport, larger particles can still achieve effective delivery due to hyaluronic acid modification, which enables receptor-mediated uptake via CD44 receptors and prolongs precorneal residence time, enhancing transcorneal permeation^32,33^. Thus, the observed sizes are suitable for ocular delivery.

### Characterization of unconjugated and PD-conjugated nanovesicles

#### Microscopic examination

TEM analysis revealed that Span 60 and Tween 80-based elastic nanovesicles formed well-defined, spherical vesicles with bilamellar to multilamellar structures. The vesicles appeared uniformly dispersed without signs of aggregation or morphological irregularities, as illustrated in Fig. 2A,B. These observations confirm the successful self-assembly of stable nanostructures using the selected surfactants.

#### FTIR spectroscopy

FTIR analysis was done to confirm the conjugation of the PD to nanovesicles, as depicted in Figure S6 (Supplementary Information). The FTIR spectrum of NVs-TM (Figure S6 A; Supplementary Information) exhibited characteristic peaks of TM and excipients. DSPE-PEG-COOH was used to functionalize NVs with carboxyl groups, and its spectrum showed peaks at 2884 and 2916 cm^−1^ (alkyl C–H), 1731 cm^−1^ (ester C=O), and 1652 cm^−1^ (acid C=O). Unconjugated PEGylated NVs (NVs-COOH) exhibited a sharp ester C=O peak at 1735 cm^−1^ and acid and amide C=O peaks at 1637 cm^−1^ and 1584 cm^−1^, respectively. Upon conjugation with PD (NVs-CONH-PD), the FTIR spectrum (Figure S6 D; Supplementary Information) showed characteristic peaks at 2849 and 2916 cm^−1^ (alkyl C–H), a less intense ester C = O peak at 1735 cm^−1^, and a new amide bond peak at 1674 cm^−1^, confirming conjugation. Broad peaks at 3355 and 3251 cm^−1^ corresponded to –NH and –OH groups. These spectral shifts confirm successful amide bond formation and conjugation of PD to the nanovesicles.

#### Differential scanning calorimetry

The DSC curves of pure TM, mannitol, NVs, and PD-NVs-TM are presented in Fig. 2F. Pure TM exhibited a prominent endothermic peak at 206 °C, which aligns with its melting point, while mannitol showed a peak at 167 °C, consistent with its known melting range (160–190 °C). In the TM-NVs formulation, the TM endothermic peak disappeared, indicating a loss of crystallinity and suggesting that TM was molecularly dispersed within the Span 60 matrix. Peaks corresponding to Span 60 and mannitol were retained at 51 °C and 158 °C, respectively. In the PD-conjugated formulation, a broad, less intense peak appeared around 115 °C, which may be attributed to the existence of the amine-terminated PD. These findings support the successful encapsulation and conjugation of TM and PD, respectively, with a notable change in thermal behavior indicating structural interactions within the nanovesicle matrix.

#### X-Ray diffraction

XRD analysis revealed characteristic crystalline peaks of TM at 14.26° and 21.46° (Figure S7 A; Supplementary Information), and a peak for Span 60 at 21.36° (Figure S7 B; Supplementary Information). The TM-Span 60 physical mixture (Figure S7 C; Supplementary Information) showed peaks from both components, though reduced TM peak intensity suggested partial amorphization. Mannitol exhibited distinct peaks at 18.74°, 23.42°, and 33.54° (Figure S7 D; Supplementary Information). Blank nanovesicles (Figure S7 E; Supplementary Information) displayed peaks corresponding to mannitol and possibly Span 60. NVs-TM (Figure S7 F; Supplementary Information) retained only a peak at 21.44°, attributed to Span 60, confirming successful drug encapsulation and transformation of TM into an amorphous or molecularly dispersed state.

#### ^1^HNMR analysis

^1^H NMR analysis was employed to verify the successful conjugation of the peptide dendrimer to the nanovesicles. As shown in Fig. 2E, the spectrum of the PD-NVs-TM exhibited additional peaks in the range of 4.0–4.5 ppm, which were absent in the spectrum of blank nanovesicles (Fig. 2D). These signals are attributed to protons present in the amino acid residues of the PD. Although the relatively low amount of PD used in the formulation limited the intensity of the signals, the appearance of these characteristic peaks confirms the presence of PD on the nanovesicle surface. These findings provide clear evidence of successful surface conjugation.

#### Determination of elasticity

The elasticity of nanovesicles is a critical parameter, as it reflects their ability to deform and penetrate the corneal membrane. The size of the optimized nanovesicles prior to extrusion was 178.9 ± 7.31 nm, and after extrusion, it measured 154.7 ± 1.23 nm. The DI of the vesicles was determined to be 1.99 ± 0.65 mL/s. These results demonstrate only a minor change (13.5%) in vesicle size, confirming their high elasticity. These results were consistent with that reported by Kakkar et al.^34^. This elasticity results from the inclusion of tween 80, which destabilizes the vesicles due to its highly flexible and non-bulky alkyl chain^22^.

#### In vitro drug release study

**Fig. 3:**
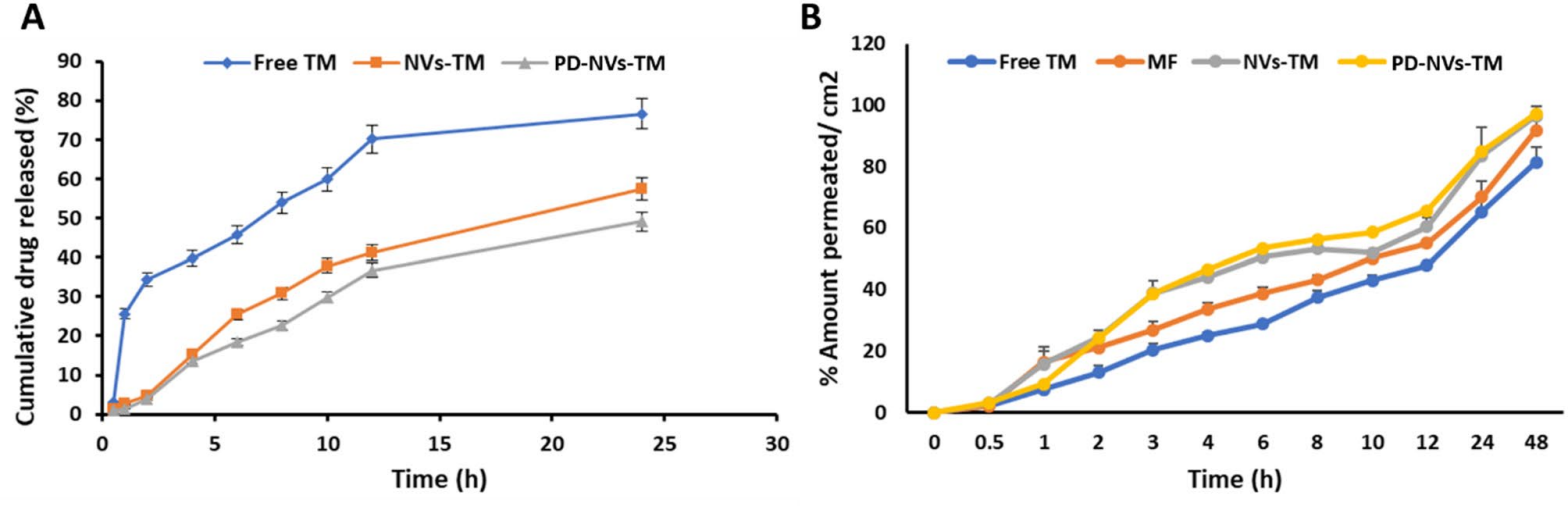
(**A**) In vitro release profile of TM from the free TM solution, NVs-TM, and PD-NVs-TM; (**B**) Comparison of cumulative drug permeation from different formulations in ex vivo corneal permeation studies using bovine cornea. All results are expressed as mean ± SD (n = 3).

In vitro dug release studies in PBS (pH 7.4) using the dialysis sac method revealed distinct release profiles for free TM and nanovesicle formulations (Fig. 3A). Free TM showed an initial burst release, with 76.55% released at 24 h. In contrast, NVs-TM exhibited sustained release, with 57.45% cumulative release over the same period. Actual concentration values at each time point are provided in Table S8; Supplementary Information. This indicates that the vesicular system effectively modulates TM release, potentially enhancing its therapeutic performance. The sustained release observed in nanovesicles can be attributed to the presence of Tween 80, which increases bilayer hydrophobicity, and Span 60, whose high phase transition temperature contributes to the formation of a more rigid and less permeable vesicular membrane^22^. PD-NVs-TM exhibited an even slower release (49.12%), likely due to surface shielding by the PD moiety, which restricts drug diffusion at physiological pH. This observation aligns with findings from Wang et al., where RGD-conjugated liposomes demonstrated slower drug release compared to unconjugated liposomes, attributed to the steric hindrance created by surface-bound ligands^35^. Such modifications can form a barrier to drug diffusion, contributing to prolonged release profiles.

#### Ex vivo corneal permeability

The results indicate that TM permeated more effectively from the nanovesicles than from the free TM solution and the marketed formulation, likely due to the nature of the nanovesicles, enhanced by the edge activator. This edge activator improves the flexibility of nanovesicles and their ability to permeate the membrane^36^. The permeability percentages obtained show a strong association with the elasticity results, which endowed the vesicles with increased membrane flexibility, enabling efficient corneal penetration^22^. Interestingly, the PD-NVs-TM formulation exhibited even higher permeation, which can be ascribed to the cationic nature of the PD. The positive charge of the peptide enables stronger interactions with the negatively charged corneal membrane, further facilitating drug permeation^37^. These findings underscore the critical role of both vesicle composition and the incorporation of specific peptides in optimizing drug delivery across the corneal barrier. The comparative corneal permeation study results of TM solution, MF, NVs-TM, and PD-NVs-TM are shown in Fig. 3B.

#### Corneal hydration

Corneal hydration is a reliable and commonly used indicator to assess the potential damage caused by a formulation to the cornea. The normal range of corneal hydration typically falls between 75 and 80%, with values exceeding 83% potentially indicating endothelial or epithelial damage^38–40^. After treatment with PD-NVs-TM, the corneal hydration was measured at 71.95% ± 1.34 (Figure S8; Supplementary Information), close to the prescribed limit, and was justified based on the lack of observed corneal damage in in vivo studies.

#### Stability study

The stability of nanovesicles was evaluated by monitoring particle size, PDI, entrapment efficiency, and aggregation during storage at 25 ± 2 °C and 4 ± 2 °C. At room temperature, the particle size increased significantly over time, suggesting potential aggregation or instability, while minimal changes were observed under refrigerated conditions, indicating better preservation. A notable 35% decrease in entrapment efficiency occurred after 30 days at room temperature, likely due to leakage or degradation, while refrigerated storage maintained entrapment efficiency. Mild aggregation was observed at room temperature after 15 and 30 days, which could compromise the formulation’s efficacy, whereas no aggregation occurred under refrigerated conditions (Table 1). This instability can be ascribed to the type of the surfactant used. In this case, Span 60, a hydrophobic surfactant, was employed, which aligns with the findings of Chatzinikoli et al**.,** They observed that hydrophobic surfactants, such as Span 80, lead to greater instability at ambient conditions compared to more hydrophilic surfactants like Tween 80, due to the more effective steric stabilization provided by hydrophilic surfactants^41^. These results highlight that refrigerated storage is optimal for maintaining the stability and integrity of elastic nanovesicles.

#### In vitro cell culture studies

##### Cellular cytotoxicity studies

**Fig. 4:**
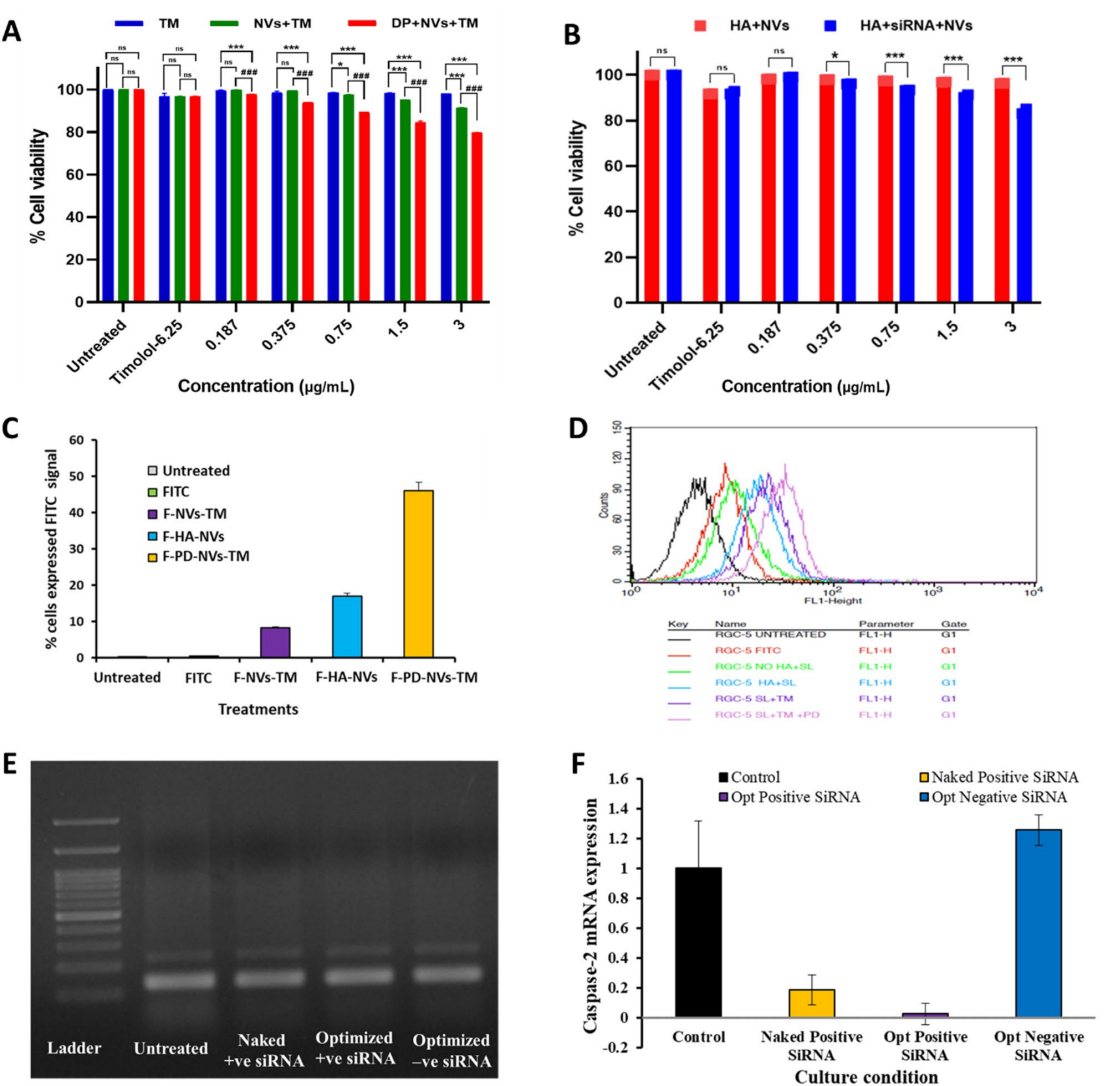
(**A**) Cell viability of HCE-2 cells after treatment with free TM, NVs-TM and PD-NVs-TM; (**B**) Cell viability of RGC-5 cells post-treatment with HA-NVs and HA-siRNA-NVs; (**C**) Quantitative FACS analysis of RGC-5 cells incubated for 24 h with untreated, FITC, FITC-labeled nanovesicles without hyaluronic acid (F-NVs-TM), FITC-labeled HA-coated nanovesicles (F-HA-NVs), and F-PD-NVs-TM; (**D**) Overlaid histograms show FITC signal intensity for each treatment group, analyzed by FACS using the FL1 channel and BD CellQuest Pro software. Caspase-2 gene expression studies; (**E**) Agarose gel electrophoresis of total RNA isolated from untreated and siRNA-transfected RGC-5 cells to assess RNA integrity ; (**F**) Relative mRNA expression of Caspase-2 genes in transfected RGC-5 cells across different culture groups, measured by RT-PCR. Results are expressed as mean ± SEM and analyzed using two-way ANOVA with (**A**) Tukey’s multiple comparison test. *P < 0.05, ***P < 0.001, ^*###*^*P* < 0.001, ns not significant. (**B**) Sidak test. ∗*p* < 0.05, ∗∗∗*p* < 0.001, ns not significant.

The cytocompatibility of various formulations was evaluated using the MTT assay. In HCE-2 cells, free TM, NVs-TM, and PD-NVs-TM showed high cell viability of 98.03%, 91.46%, and 79.81%, respectively at the highest tested concentration (3 mg/mL), indicating good biocompatibility (Fig. 4A). Similarly, RGC-5 cells treated with HA-NVs and HA-siRNA-NVs exhibited 96.16% and 83.01% viability, respectively, confirming low cytotoxicity (Fig. 4B). Morphology of HCE-2 AND RGC-5 cells after treatment with various formulations is shown in Figure S9-S10; Supplementary Information.

##### Cellular uptake studies

To elucidate the interaction of nanovesicles with Retinal Ganglion Cells (RGC-5) and the effect of PD functionalization on the nanovesicles, the internalization of FITC-labeled nanovesicles was studied in vitro. The cellular uptake of free FITC dye and FITC-labeled nanovesicles (F-NVs-TM), HA-coated nanovesicles, and PD-NVs-TM in RGC-5 cells was assessed using flow cytometry after 24 h (Fig. 4C). The nanovesicles exhibited higher fluorescence intensity than free FITC dye, indicating better cellular uptake. This result aligns with the work of Kakkar et al., who demonstrated that 6-carboxyfluorescein-labeled nanovesicles were effectively internalized in eye tissues, highlighting their potential for drug delivery to the posterior segments of the eye^34^. The internalization mechanism in retinal ganglion cells is likely due to the vesicles’ ability to squeeze through the cornea and interact with the aqueous and vitreous humor. Among the formulations, PD-NVs-TM showed the highest cellular uptake, likely due to the dendrimer’s ability to facilitate internalization through electrostatic attraction with the negatively charged cell membrane^42^. Additionally, the dendrimers can disrupt the lipid bilayer and loosen epithelial cell junctions, further aiding vesicle entry^43^. The phospholipids in the cell membrane also form hydrogen bonds with the guanidine functional group of arginine, contributing to the internalization of the nanovesicles^44^. The overlaid histogram, representing the intensity of the FITC signal of untreated and treated cells, is shown in Fig. 4D.

Furthermore, HA-coated nanovesicles showed enhanced uptake compared to uncoated ones, consistent with studies by Huang et al. Their study showed that coating nanoparticles with HA enhances cellular uptake and promotes better penetration of retinal tissue ex vivo. This enhanced uptake is linked to the specific interaction of HA with CD44 receptors, facilitating targeted delivery to retinal cells. In the retina, CD44 is a commonly expressed transmembrane glycoprotein located on retinal pigment epithelial cells, Müller cells, and retinal ganglion cells, and serves as a receptor for HA^45^. HA, a natural component of the vitreous body and aqueous humor, enhances drug residence time and bioavailability by forming non-covalent bonds with the mucin layer of the cornea. Specifically, the carboxyl groups of HA interact with the sialic acid residues in ocular mucin, facilitating adhesion and improving drug retention at the site of action^46^. Moreover, HA binds to CD44 receptors on retinal cells, promoting nanoparticle uptake and penetration into retinal tissue^47^. These findings suggest that HA-coated nanovesicles may effectively target retinal cells, enhancing their potential for ocular drug delivery.

##### Caspase-2 gene expression studies

RNA was quantified using UV–Vis spectroscopy with the QiaExpert (Qiagen, CA). The RNA purity was assessed by calculating A_260/280_ ratio, which was close to 2.0 (Table S10; Supplementary Information), indicating that the RNA was pure^48^. To further assess RNA integrity, 2 µg of RNA was run on a 1.5% agarose gel alongside a Lambda HindIII/EcoRI (100 bp) ladder. Both the 18S and 28S ribosomal RNA bands were visible in the control and siRNA-treated groups (Fig. 4E), confirming the RNA’s integrity. Gene expression was analyzed using raw fluorescence data (Ct values) from the real-time PCR instrument (Qiagen), which was exported to Rotor-Gene Q software (version 2.3.4). Data were normalized to beta-actin using the formula:

$$\Delta {\text{Ct}} = {\text{Average}}\,{\text{Ct}}\,{\text{of}}\,{\text{test}}\,{\text{sample}} - {\text{Average}}\,{\text{Ct}}\,{\text{of}}\,{\text{calibrator}}.$$

Relative Quantification represents the fold change relative to the calibrator, values exceeding 1 indicate upregulation, whereas values below 1 suggest downregulation. Gene expression fold change was assessed by comparing the Ct values in both the treated and untreated samples. The results depicted that the *Caspase-2* gene expression was downregulated in both the Optimized Positive siRNA and Naked Positive siRNA treatment groups, while the Optimized Negative siRNA group exhibited upregulation compared to the untreated group. Beta-actin served as the internal control in this study. The findings (Table S11; Supplementary Information) and (Fig. 4F) confirm that the Optimized Positive siRNA formulation effectively inhibited Caspase-2 gene expression in transfected RGC-5 cells.

#### In vivo safety and efficacy studies

##### Eye irritation studies

**Fig. 5:**
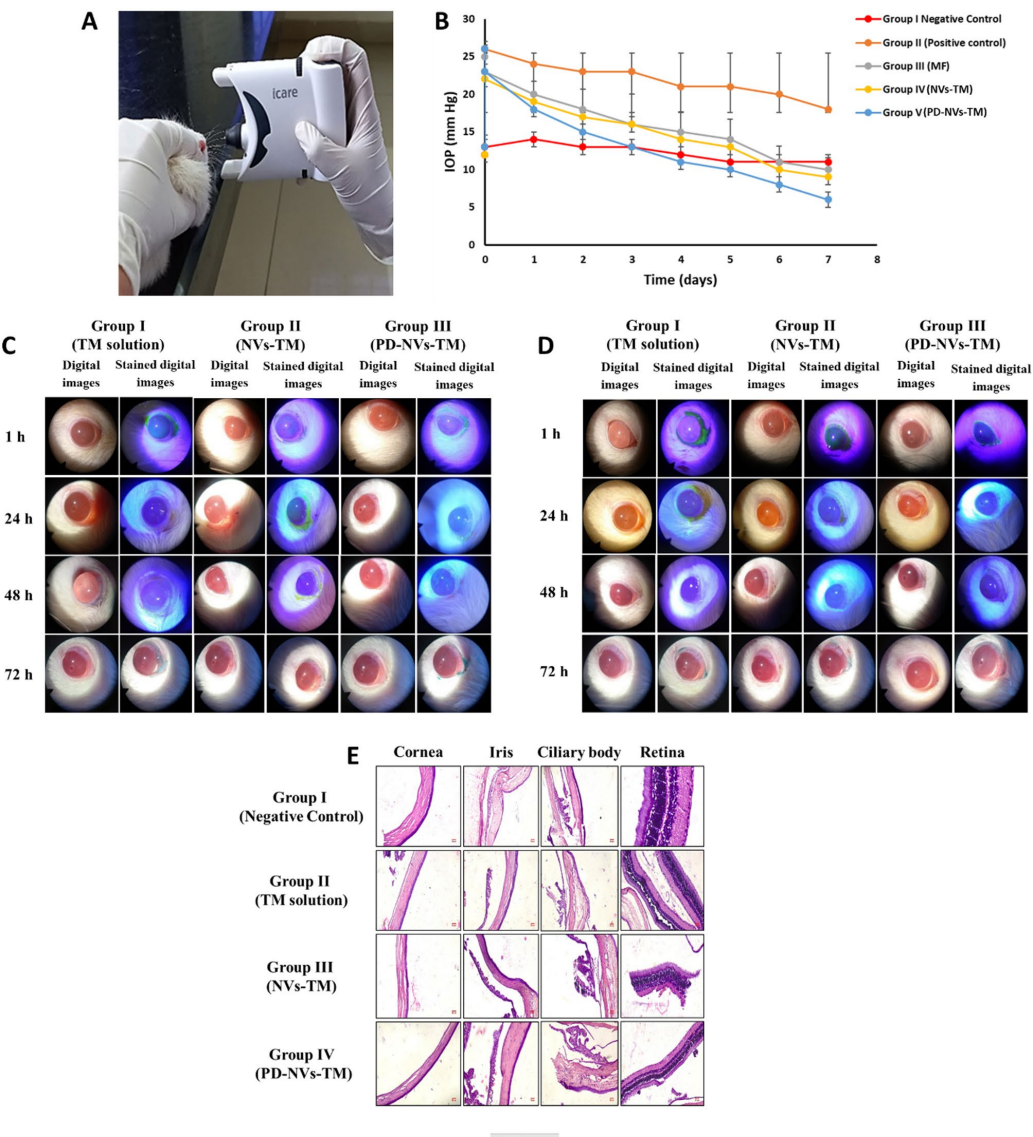
(**A**) Image showing IOP measurement in an SD rat using the iCare Home Tonometer. (**B**) IOP changes (mmHg, mean ± SD, n = 3) at different time points post-administration across the following groups: untreated control (Group I), normal saline (Group II), marketed formulation (Group III), NVs-TM (Group IV), and PD-NVs-TM (Group V). Microscopic images of rat eyes treated with 0.1 mL of 0.5% TM solution (Group I), NVs (Group II), PD-NVs-TM (Group III) for (**C**) Acute eye irritation and (**D**) Chronic eye irritation. (**E**) Haematoxylin and Eosin stained images of rat cornea, iris, ciliary body, and retina after 1 week (100x), showing results for negative control (Group I), 0.5% TM solution (Group II), NVs (Group III) and PD-NVs-TM (Group IV).

The in vivo acute eye irritation study was conducted using a 0.5% w/v TM solution, NVs-TM, and PD-NVs-TM. Ocular irritation scores for the cornea, iris, conjunctiva, and chemosis were recorded (Table S12; Supplementary Information), based on microscopic observations (Fig. 5C). All formulations met the criteria for no irritation in the cornea and iris. However, mild irritation of the conjunctiva and slight eyelid swelling were observed 24 h after administration of the 0.5% w/v TM solution. Similarly, the in vivo chronic eye irritation study was performed with the same formulations. Ocular irritation scores were again recorded for the cornea, iris, conjunctiva, and chemosis (Table S13; Supplementary Information), and the corresponding microscopic images are shown in Fig. 5D. The results confirmed no irritation in the iris for any of the formulations, but mild irritation of the cornea and conjunctiva, as well as slight eyelid swelling, was noted after treatment with the NVs-TM.

To assess corneal epithelial integrity, sodium fluorescein was used to detect any breaks at 1, 24, and 48 h post-treatment. Stained digital images revealed no ocular damage with any of the formulations. Post 72 h, lissamine green staining was performed to evaluate potential cell degeneration or cell death. No premature cell death or degeneration was observed, indicating that the formulations were non-toxic and well-tolerated. These observations indicate that the optimized formulations are safe and non-irritating. Similar findings have been reported in previous studies, where nano-formulations, were shown to have minimal ocular irritation when compared to conventional formulations^49,50^.

##### Pharmacodynamics study

Glaucoma was induced in SD rats by episcleral vein injection of hypertonic saline, resulting in a significant increase in IOP from a baseline of 13–26 mm Hg. IOP was monitored daily using an iCare home tonometer (Fig. 5A), and various formulations were tested. In the positive control group (Group II), where rats received normal saline, no reduction in IOP was observed, and it remained elevated over the 7-day treatment period. Group III, treated with commercial TM eye drops showed a significant reduction in IOP from 25 to 10 mm Hg, validating the anti-glaucoma effect of TM. Group IV, treated with NVs-TM, demonstrated an even more pronounced IOP reduction, from 23 to 9 mm Hg, suggesting the nanovesicles facilitated better corneal penetration and sustained drug release.

The most significant reduction in IOP was observed in Group V, where rats received PD-NVs-TM, with IOP decreasing from 26 to 6 mm Hg. This enhanced effect is attributed to the PD conjugation, which improves corneal permeation. The cationic nature of dendrimers enables better interaction with the negatively charged corneal membranes, thereby enhancing drug delivery^28^. Previous studies have shown that dendrimer-based systems, such as DenTimol, can efficiently cross the cornea and provide sustained therapeutic effects without ocular irritation^51^. Table S14; Supplementary Information summarizes the mean IOP values, and Fig. 5B compares the IOP-lowering effects observed in the different treatment groups. In conclusion, PD-conjugated nanovesicles offer superior IOP-lowering effects compared to traditional eye drops due to their enhanced corneal permeation, sustained drug release, and overall therapeutic efficacy.

##### Histopathological examination

The corneal tissue in all treatment groups retained normal architecture, with clearly distinguishable endothelial, stromal, and elastic layers. No evidence of inflammatory infiltration or neovascularization was observed, except in NVs-TM, which exhibited corneal epithelial hyperplasia accompanied by acute inflammatory cell infiltration and neovascularization. In contrast, PD-NVs-TM did not show any pathological changes, indicating improved biocompatibility. Similarly, histological examination of the iris, ciliary body, and retina revealed no signs of structural damage, atrophy, vacuolation, or inflammation across all groups. The cellular organization and tissue morphology remained intact. These findings suggest that the tested formulations, particularly the PD-NVs-TM, demonstrated favourable corneal biocompatibility and were non-irritant to ocular tissues. The grading of corneal histopathological changes is summarized in Table S15; Supplementary Information, while representative histological images are presented in Fig. 5E.

## Conclusion

This study presents an innovative dual-delivery approach for glaucoma management using Spanlastic nanovesicles, with timolol maleate and Caspase-2 siRNA encapsulated in separate but complementary formulations. TM-loaded nanovesicles were conjugated with a peptide dendrimer to enhance corneal permeation, while siRNA-loaded nanovesicles were coated with hyaluronic acid to facilitate targeted delivery to RGC. Both formulations exhibited favourable physicochemical characteristics, indicating good stability and suitability for ocular administration. Physicochemical evaluations further confirmed high drug encapsulation efficiency and sustained release profiles. In vitro and ex vivo studies demonstrated efficient drug delivery and enhanced transcorneal permeation, while cytotoxicity assays established the safety and biocompatibility of the formulations. The PD-NVs-TM formulation demonstrated significantly enhanced corneal penetration and achieved sustained intraocular pressure reduction in in vivo pharmacodynamic studies. In parallel, the siRNA-loaded spanlastics effectively silenced the Caspase-2 gene and prevented apoptosis in RGC, suggesting a potential neuroprotective effect, which warrants further exploration. Histopathological evaluation and ocular irritation studies further confirmed that both formulations were non-irritant and well-tolerated, with no signs of tissue damage or inflammatory response. Collectively, these findings highlight the promise of a dual-spanlastic-based delivery platform as a safe, non-invasive, and effective therapeutic strategy for glaucoma, combining IOP-lowering efficacy with targeted *caspase-2* silencing in RGC.

## Electronic supplementary material

Below is the link to the electronic supplementary material.

Supplementary Material 1

## Data Availability

Data is presented in the main manuscript and Supplementary information.

## References

[1] Danesh-Meyer, H. V. & Levin, L. A. Glaucoma as a neurodegenerative disease. *J. Neuroophthalmol.* **35**, S22 (2015). 26274833 10.1097/WNO.0000000000000293

[2] Shan, S. et al. Global incidence and risk factors for glaucoma: A systematic review and meta-analysis of prospective studies. *J. Glob. Health* **14**, 04252 (2024). 39513294 10.7189/jogh.14.04252 PMC11544525

[3] Tham, Y.-C. et al. Global prevalence of glaucoma and projections of glaucoma burden through 2040: A systematic review and meta-analysis. *Ophthalmology* **121**, 2081–2090 (2014). 24974815 10.1016/j.ophtha.2014.05.013

[4] Gautam, D., Talwan, P., Chaurasia, H., Kumar, S. & Singh, R. Nanotechnology carriers for the management, electrochemical detection and diagnosis of glaucoma. In *Electrocatalytic Materials* (eds Patra, S. et al.) 527–559 (Springer, 2024). 10.1007/978-3-031-65902-7_15.

[5] Sah, A. K. & Suresh, P. K. Medical management of glaucoma: focus on ophthalmologic drug delivery systems of timolol maleate. *Artif. Cells Nanomed. Biotechnol.* **45**, 448–459 (2017). 27002850 10.3109/21691401.2016.1160917

[6] Volotinen, M., Hakkola, J., Pelkonen, O., Vapaatalo, H. & Mäenpää, J. Metabolism of ophthalmic timolol: New aspects of an old drug. *Basic Clin. Pharmacol. Toxicol.* **108**, 297–303 (2011). 21385322 10.1111/j.1742-7843.2011.00694.x

[7] Cuggino, J. C., Tártara, L. I., Gugliotta, L. M., Palma, S. D. & Alvarez Igarzabal, C. I. Mucoadhesive and responsive nanogels as carriers for sustainable delivery of timolol for glaucoma therapy. *Mater. Sci. Eng. C* **118**, 111383 (2021). 10.1016/j.msec.2020.111383 33254990

[8] Muhtadi, W. K., Novitasari, L., Danarti, R. & Martien, R. Development of polymeric nanoparticle gel prepared with the combination of ionic pre-gelation and polyelectrolyte complexation as a novel drug delivery of timolol maleate. *Drug Dev. Ind. Pharm.* **46**, 1844–1852 (2020). 32901561 10.1080/03639045.2020.1821053

[9] Wu, K. Y., Ashkar, S., Jain, S., Marchand, M. & Tran, S. D. Breaking barriers in eye treatment: polymeric nano-based drug-delivery system for anterior segment diseases and glaucoma. *Polymers* **15**, 1373 (2023). 36987154 10.3390/polym15061373 PMC10054733

[10] Levkovitch-Verbin, H. Retinal ganglion cell apoptotic pathway in glaucoma: Initiating and downstream mechanisms. *Prog. Brain Res.* **220**, 37–57 (2015). 26497784 10.1016/bs.pbr.2015.05.005

[11] Sarkis, S., Chamard, C., Johansen, B., Daien, V. & Michon, F. Challenging glaucoma with emerging therapies: an overview of advancements against the silent thief of sight. *Front. Med.* **12**, 1527319 (2025). 10.3389/fmed.2025.1527319 PMC11979169 40206485

[12] Vigneswara, V., Berry, M., Logan, A. & Ahmed, Z. Pharmacological inhibition of caspase-2 protects axotomised retinal ganglion cells from apoptosis in adult rats. *PLoS ONE* **7**, e53473 (2012). 23285297 10.1371/journal.pone.0053473 PMC3532067

[13] Solano, E. C. R. et al. Toxicological and pharmacokinetic properties of QPI-1007, a chemically modified synthetic siRNA targeting caspase 2 mRNA, following intravitreal injection. *Nucleic Acid Ther.* **24**, 258–266 (2014). 25054518 10.1089/nat.2014.0489

[14] Mutalik, S. et al. Development and validation of a reversed-phase high-performance liquid chromatographic method for quantification of peptide dendrimers in human skin permeation experiments. *J. Chromatogr. B* **877**, 3556–3562 (2009). 10.1016/j.jchromb.2009.08.039 19744897

[15] Shahrukh, S. et al. Quality by design enabled tumor microenvironment-responsive simvastatin-loaded liposomes for prostate cancer management. *J. Drug Deliv. Sci. Technol.* **94**, 105474 (2024).

[16] Bagul, U. S. et al. Fabrication of acetazolamide loaded leciplex for intraocular delivery: Optimization by 32 full factorial design, *in vitro, ex vivo* and *in vivo* pharmacodynamics. *Int. J. Pharm.* **661**, 124391 (2024). 38936444 10.1016/j.ijpharm.2024.124391

[17] Liu, Y., Wang, Y., Yang, J., Zhang, H. & Gan, L. Cationized hyaluronic acid coated spanlastics for cyclosporine A ocular delivery: Prolonged ocular retention, enhanced corneal permeation and improved tear production. *Int. J. Pharm.* **565**, 133–142 (2019). 31075435 10.1016/j.ijpharm.2019.05.018

[18] El-Meshad, A. N. & Mohsen, A. M. Enhanced corneal permeation and antimycotic activity of itraconazole against Candida albicans via a novel nanosystem vesicle. *Drug Deliv.* **23**, 2115–2123 (2016). 25080226 10.3109/10717544.2014.942811

[19] Li, H. et al. Liposomes as a novel ocular delivery system for brinzolamide. in vitro and in vivo studies. *AAPS PharmSciTech* **17**, 710–717 (2016). 26335415 10.1208/s12249-015-0382-1

[20] Naik, S. et al. Full factorial design for development and validation of a stability-indicating RP-HPLC method for the estimation of timolol maleate in surfactant-based elastic nano-vesicular systems. *J. Chromatogr. Sci.* **60**, 584–594 (2022). 34435614 10.1093/chromsci/bmab101

[21] Huang, J. et al. Ocular cubosome drug delivery system for timolol maleate: Preparation, characterization, cytotoxicity, ex vivo, and in vivo evaluation. *AAPS PharmSciTech* **18**, 2919–2926 (2017). 28429294 10.1208/s12249-017-0763-8

[22] Abdelbari, M. A., El-mancy, S. S., Elshafeey, A. H. & Abdelbary, A. A. Implementing spanlastics for improving the ocular delivery of clotrimazole: In vitro characterization, ex vivo permeability, microbiological assessment and in vivo safety study. *Int. J. Nanomed.* **16**, 6249–6261 (2021). 10.2147/IJN.S319348 PMC8439980 34531656

[23] Luechtefeld, T. et al. Analysis of Draize eye irritation testing and its prediction by mining publicly available 2008–2014 REACH data. *Altex* **33**, 123–134 (2016). 26863293 10.14573/altex.1510053 PMC5461467

[24] Xu, B., Jacobs, M. I., Kostko, O. & Ahmed, M. Guanidinium group remains protonated in a strongly basic arginine solution. *ChemPhysChem* **18**, 1503–1506 (2017). 28231411 10.1002/cphc.201700197

[25] Liu, C. et al. Arginine-terminated generation 4 PAMAM dendrimer as an effective nanovector for functional sirna delivery in vitro and in vivo. *Bioconjug. Chem.* **25**, 521–532 (2014). 24494983 10.1021/bc4005156

[26] Thompson, M. & Scholz, C. Highly branched polymers based on poly(amino acid)s for biomedical application. *Nanomaterials* **11**, 1119 (2021). 33925961 10.3390/nano11051119 PMC8145254

[27] Manikkath, J., Parekh, H. S. & Mutalik, S. Surface-engineered nanoliposomes with lipidated and non-lipidated peptide-dendrimeric scaffold for efficient transdermal delivery of a therapeutic agent: Development, characterization, toxicological and preclinical performance analyses. *Eur. J. Pharm. Biopharm.* **156**, 97–113 (2020). 32911066 10.1016/j.ejpb.2020.09.001

[28] Manikkath, J., Hegde, A. R., Kalthur, G., Parekh, H. S. & Mutalik, S. Influence of peptide dendrimers and sonophoresis on the transdermal delivery of ketoprofen. *Int. J. Pharm.* **521**, 110–119 (2017). 28163223 10.1016/j.ijpharm.2017.02.002

[29] Hegde, A. R. et al. Peptide dendrimer-conjugates of ketoprofen: synthesis and ex vivo and in vivo evaluations of passive diffusion, sonophoresis and iontophoresis for skin delivery. *Eur. J. Pharm. Sci.* **102**, 237–249 (2017). 28285173 10.1016/j.ejps.2017.03.009

[30] Wang, X. & Gao, Y. Effects of length and unsaturation of the alkyl chain on the hydrophobic binding of curcumin with Tween micelles. *Food Chem.* **246**, 242–248 (2018). 29291845 10.1016/j.foodchem.2017.11.024

[31] El Zaafarany, G. M., Awad, G. A. S., Holayel, S. M. & Mortada, N. D. Role of edge activators and surface charge in developing ultradeformable vesicles with enhanced skin delivery. *Int. J. Pharm.* **397**, 164–172 (2010). 20599487 10.1016/j.ijpharm.2010.06.034

[32] Wadhwa, S., Paliwal, R., Paliwal, S. R. & Vyas, S. P. Hyaluronic acid modified chitosan nanoparticles for effective management of glaucoma: Development, characterization, and evaluation. *J. Drug Target* **18**, 292–302 (2010). 19943753 10.3109/10611860903450023

[33] Silva, M. M. et al. Chitosan nanoparticles as a mucoadhesive drug delivery system for ocular administration. *Mar. Drugs* **15**, 370 (2017). 29194378 10.3390/md15120370 PMC5742830

[34] Kakkar, S. & Kaur, I. P. Spanlastics—A novel nanovesicular carrier system for ocular delivery. *Int. J. Pharm.* **413**, 202–210 (2011). 21540093 10.1016/j.ijpharm.2011.04.027

[35] Wang, F., Chen, L., Zhang, R., Chen, Z. & Zhu, L. RGD peptide conjugated liposomal drug delivery system for enhance therapeutic efficacy in treating bone metastasis from prostate cancer. *J. Control. Release* **196**, 222–233 (2014). 25456829 10.1016/j.jconrel.2014.10.012

[36] Bayindir, Z. S. & Yuksel, N. Characterization of niosomes prepared with various nonionic surfactants for paclitaxel oral delivery. *J. Pharm. Sci.* **99**, 2049–2060 (2010). 19780133 10.1002/jps.21944

[37] Kwok, A. et al. Efficient transfection of siRNA by peptide dendrimer-lipid conjugates. *ChemBioChem* **17**, 2223–2229 (2016). 27862758 10.1002/cbic.201600485

[38] Ahuja, M., Dhake, A. S. & Majumdar, D. K. Effect of formulation factors on in vitro permeation of diclofenac from experimental and marketed aqueous eye drops through excised goat cornea. *Yakugaku Zasshi* **126**, 1369–1375 (2006). 17139162 10.1248/yakushi.126.1369

[39] Huang, D., Chen, Y.-S. & Rupenthal, I. D. Hyaluronic acid coated albumin nanoparticles for targeted peptide delivery to the retina. *Mol. Pharm.* **14**, 533–545 (2017). 27997199 10.1021/acs.molpharmaceut.6b01029

[40] Zafar, A. et al. Formulation of carteolol chitosomes for ocular delivery: formulation optimization, ex-vivo permeation, and ocular toxicity examination. *Cutan. Ocul. Toxicol.* **40**, 338–349 (2021). 34340615 10.1080/15569527.2021.1958225

[41] Chatzinikoli, L., Pippa, N. & Demetzos, C. Preparation and physicochemical characterization of elastic liposomes: a road-map library for their design. *J. Liposome Res.* **31**, 11–18 (2021). 31631722 10.1080/08982104.2019.1682605

[42] Rewatkar, P. V., Parekh, H. S. & Parat, M.-O. Molecular determinants of the cellular entry of asymmetric peptide dendrimers and role of caveolae. *PLoS ONE* **11**, e0147491 (2016). 26788849 10.1371/journal.pone.0147491 PMC4720277

[43] Yao, W.-J. et al. Effect of poly (amidoamine) dendrimers on corneal penetration of puerarin. *Biol. Pharm. Bull.* **33**, 1371–1377 (2010). 20686234 10.1248/bpb.33.1371

[44] Futaki, S. Membrane-permeable arginine-rich peptides and the translocation mechanisms. *Adv. Drug Deliv. Rev.* **57**, 547–558 (2005). 15722163 10.1016/j.addr.2004.10.009

[45] Clark, S. J. et al. Mapping the differential distribution of glycosaminoglycans in the adult human retina, choroid, and sclera. *Invest. Ophthalmol. Vis. Sci.* **52**, 6511–6521 (2011). 21746802 10.1167/iovs.11-7909 PMC3175996

[46] Zhang, X., Wei, D., Xu, Y. & Zhu, Q. Hyaluronic acid in ocular drug delivery. *Carbohydr. Polym.* **264**, 118006 (2021). 33910737 10.1016/j.carbpol.2021.118006

[47] Guter, M. & Breunig, M. Hyaluronan as a promising excipient for ocular drug delivery. *Eur. J. Pharm. Biopharm.* **113**, 34–49 (2017). 27914235 10.1016/j.ejpb.2016.11.035

[48] Wilfinger, W. W., Mackey, K. & Chomczynski, P. Effect of pH and ionic strength on the spectrophotometric assessment of nucleic acid purity. *Biotechniques* **22**, 474–481 (1997). 9067025 10.2144/97223st01

[49] Mohammad, et al. Topical tacrolimus progylcosomes nano-vesicles as a potential therapy for experimental dry eye syndrome. *J. Pharm. Sci.* **111**, 479–484 (2022). 34599998 10.1016/j.xphs.2021.09.038

[50] Wang, T. et al. A topical fluorometholone nanoformulation fabricated under aqueous condition for the treatment of dry eye. *Colloids Surf. B Biointerfaces* **212**, 112351 (2022). 35091382 10.1016/j.colsurfb.2022.112351

[51] Lancina, M. G. I., Wang, J., Williamson, G. S. & Yang, H. Dentimol as a dendrimeric timolol analogue for glaucoma therapy: synthesis and preliminary efficacy and safety assessment. *Mol. Pharm.* **15**, 2883–2889 (2018). 29767982 10.1021/acs.molpharmaceut.8b00401 PMC6075655

