# Supplementary material for: Peptide dendrimer and hyaluronic acid modified nanovesicles for ocular delivery of timolol maleate and siRNA
Source: Sci Rep. 2025 Jul 18;15:26074. doi: 10.1038/s41598-025-10960-9 (PMC12274417; doi:10.1038/s41598-025-10960-9)
Supplement: Supplementary file 1 — Supplementary Material 1 [file 41598_2025_10960_MOESM1_ESM.docx]

**Peptide Dendrimer and Hyaluronic Acid Modified Nanovesicles for Ocular Delivery of Timolol Maleate and siRNA**

Santoshi Naik^1^, Naitik Jain^1^, Nagarajan Theruveethi^2^, Srinivas Mutalik^1*^

^1^ Department of Pharmaceutics, Manipal College of Pharmaceutical Sciences, Manipal Academy of Higher Education, Manipal 576104, Karnataka, India

^2^ Department of Optometry, Manipal College of Health Professions, Manipal Academy of Higher Education, Manipal 576104, Karnataka, India

***Corresponding Author**

Dr Srinivas Mutalik

Professor and Principal

Manipal College of Pharmaceutical Sciences

Manipal Academy of Higher Education

Manipal 576104, Karnataka, India

Email: [ss.mutalik@manipal.edu](mailto:ss.mutalik@manipal.edu)

**Section S1**

**Drug - Excipient compatibility studies**

The FTIR spectrum of the physical mixture containing TM, Span 60, and Tween 80 (**Figure S1 C)** exhibited the characteristic peaks of the drug at 3491 cm⁻¹ (O-H stretching of carboxylic acid), 2919 cm⁻¹ (C-H stretching of methyl groups), 1734 cm⁻¹ (C=O stretching of ketone), 1640 cm⁻¹ (C=C stretching in the aromatic ring), 1457 cm⁻¹ (aliphatic C-H bending), 1293 cm⁻¹ (aliphatic C-N stretching), 944 cm⁻¹ (N-H wagging), and 850 cm⁻¹ (C-H bending). Comparison with the spectra of Span 60 (**Figure S1 A**) and Tween 80 (**Figure S1 B)** confirmed that these peaks remained unaffected in the mixture, indicating no significant chemical interaction or incompatibility between the drug and the excipients.

To further confirm this compatibility, DSC analysis was conducted **(Figure S1 D)**. Pure TM exhibited a sharp endothermic peak at 206°C, corresponding to its melting point and indicating its crystalline nature, while Span 60 showed a peak at 54°C. In the physical mixture, the TM peak shifted slightly to 196°C, and Span 60 melted at 55°C. The reduced intensity of TM’s endothermic peak suggests partial solubilization in molten Span 60 before reaching its melting point. The minor decrease in TM’s melting temperature is likely due to the moistening effect of the excipient, further supporting the absence of any significant interaction and confirming the chemical stability of the drug in the presence of Span 60.

**Figures**


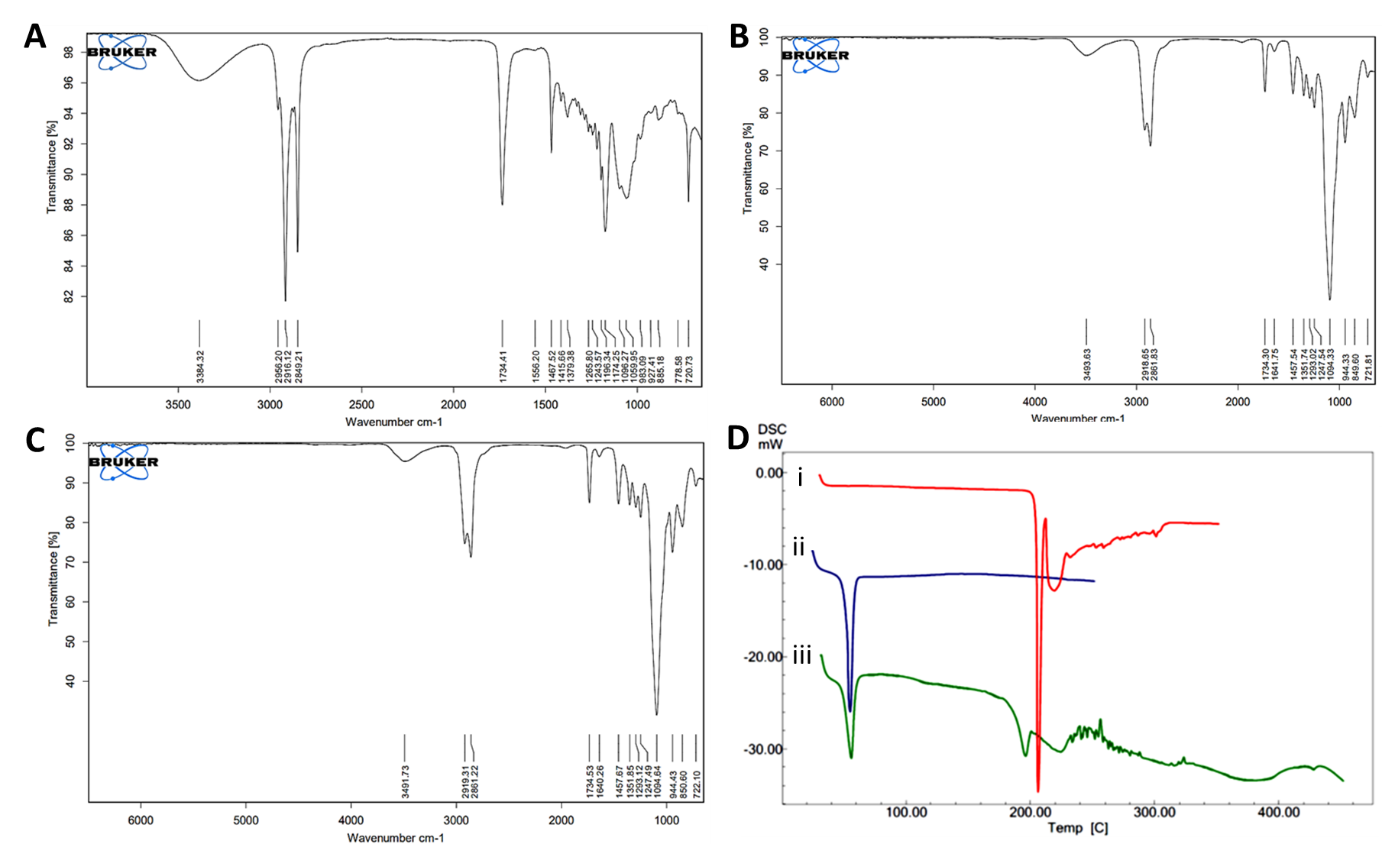


**Figure S1. FTIR spectra of (A) Span 60 (B) Tween 80 (C) Physical mixture of TM, span 60 and tween 80. (D) DSC curve of i) TM ii) Span 60 iii) Physical mixture of TM and Span 60.**


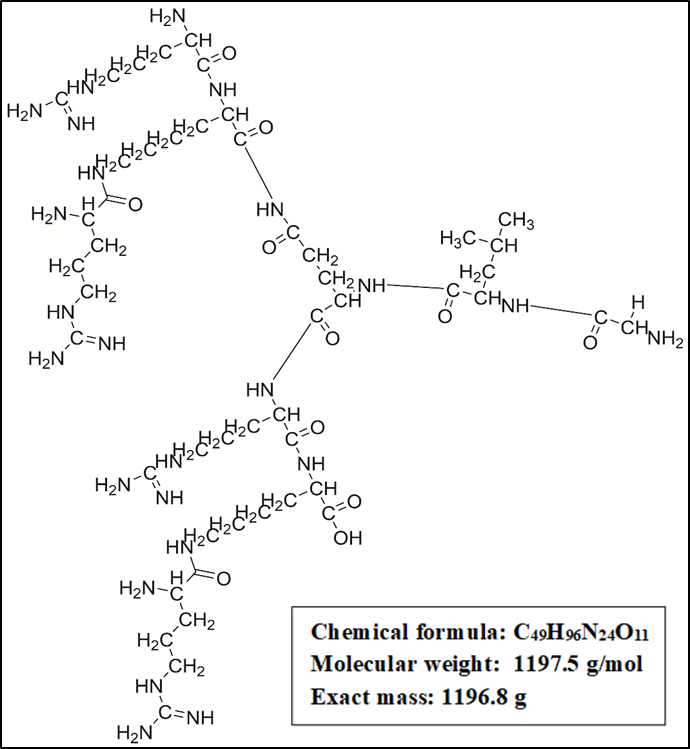


**Figure S2. Structure of peptide dendrimer**

**
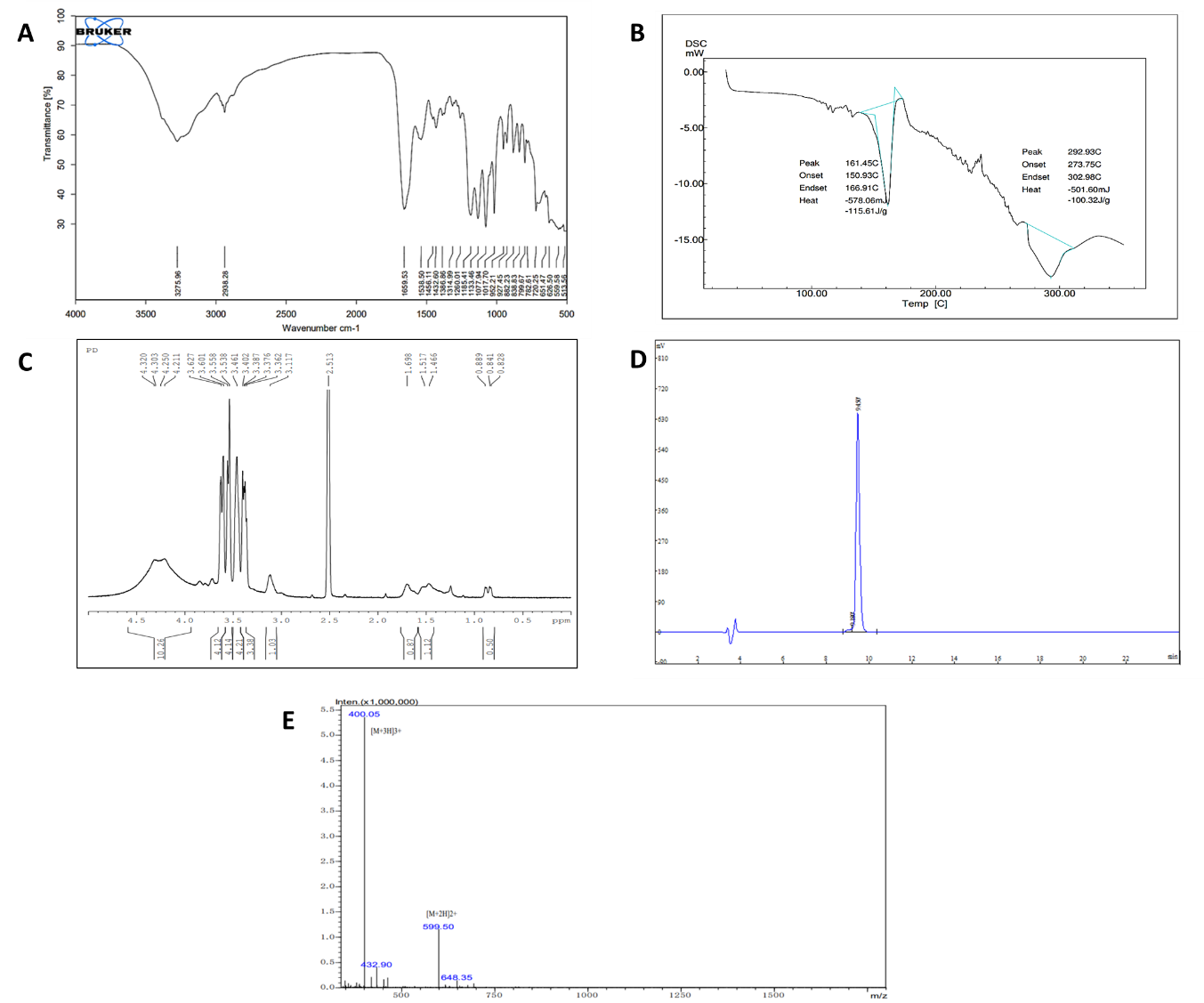
**

**Figure S3. Characterization of Peptide dendrimer (A) FTIR spectra, (B) DSC graph, (C) ^1^H-NMR spectrum, (D) HPLC chromatogram, (E) Mass spectra.**

**
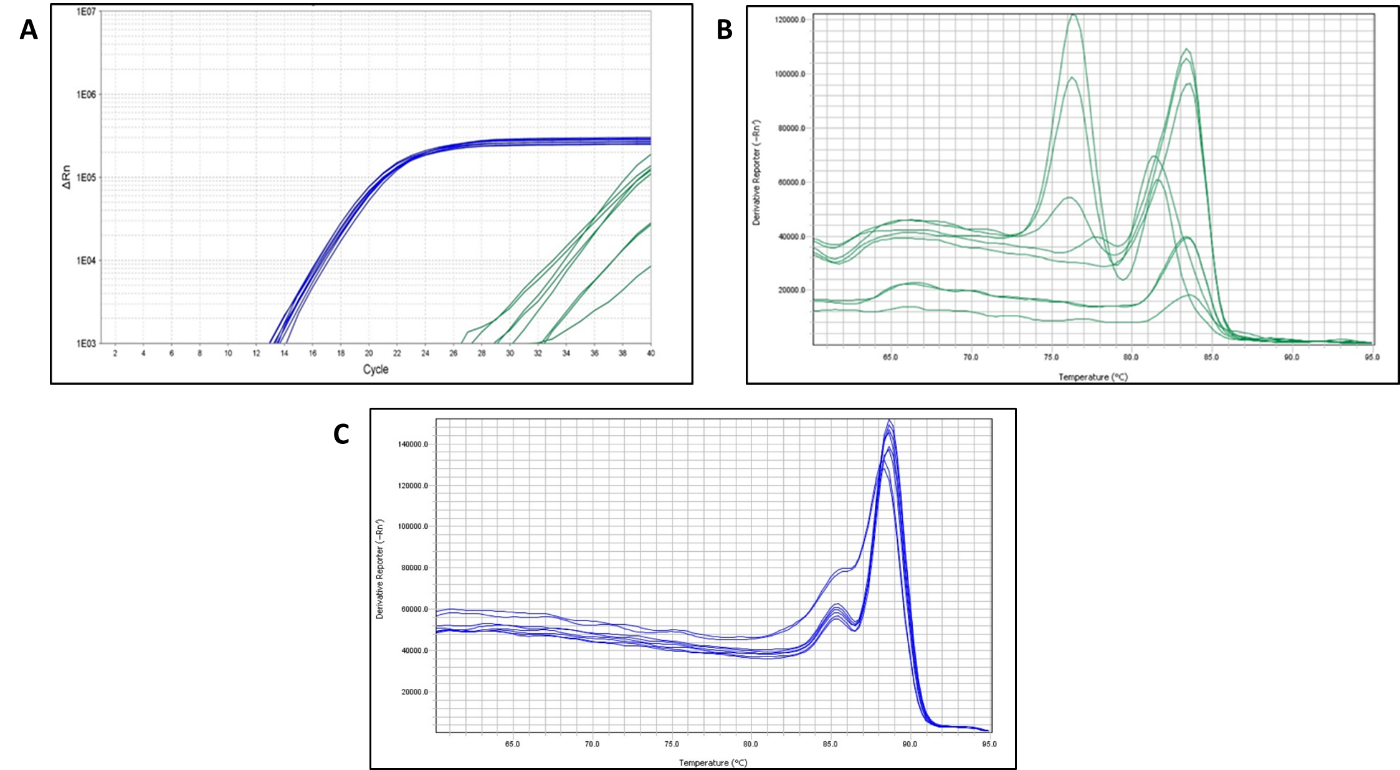
Figure S4. Validation of qPCR primers. (A) Amplification plot showing the validation of designed primers with each cDNA, demonstrating efficient amplification. (B) Melting curve analysis of the *Caspase-2* gene, confirming product specificity. (C) Melting curve analysis of the *Beta-actin* gene.**

The sequence of the designed siRNA is as mentioned below:

Sense strand 5' ACCUCCUAGAGAAGGACAU [dT][dT]3'

Antisense strand 5' AUGUCCUUCUCUAGGAGGU [dT][dT]3'

The sequences of primer are as mentioned below:

1. **Caspase-2**

Forward Sequence 5′-TCATCCAAGCATGTCGTGGAGG-3′

Reverse Sequence 5′-GCAGTGAACAGAAGGAGGTGCC-3′

**2. Beta-actin**

Forward Sequence 5′-GATCATTGCTCCTCCTGAGC -3′

Reverse Sequence 5′-AGTCCGCCTAGAAGCACTTG -3′

**
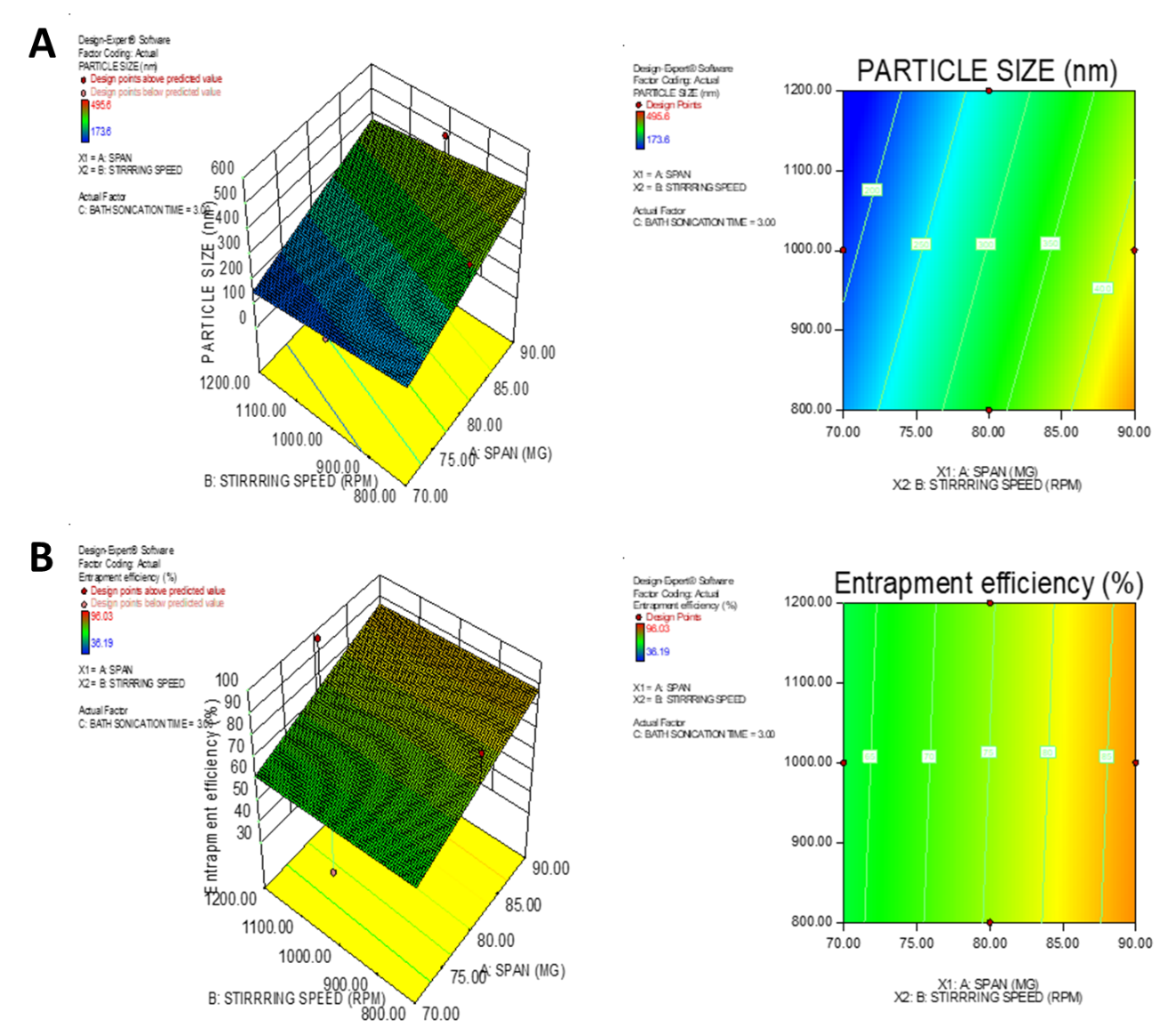
**

**Figure S5:** **3D effect surface plot and contour plot illutrating the interaction between concentration of span and stirring speed on (A) particle size (B) entrapment efficiency.**


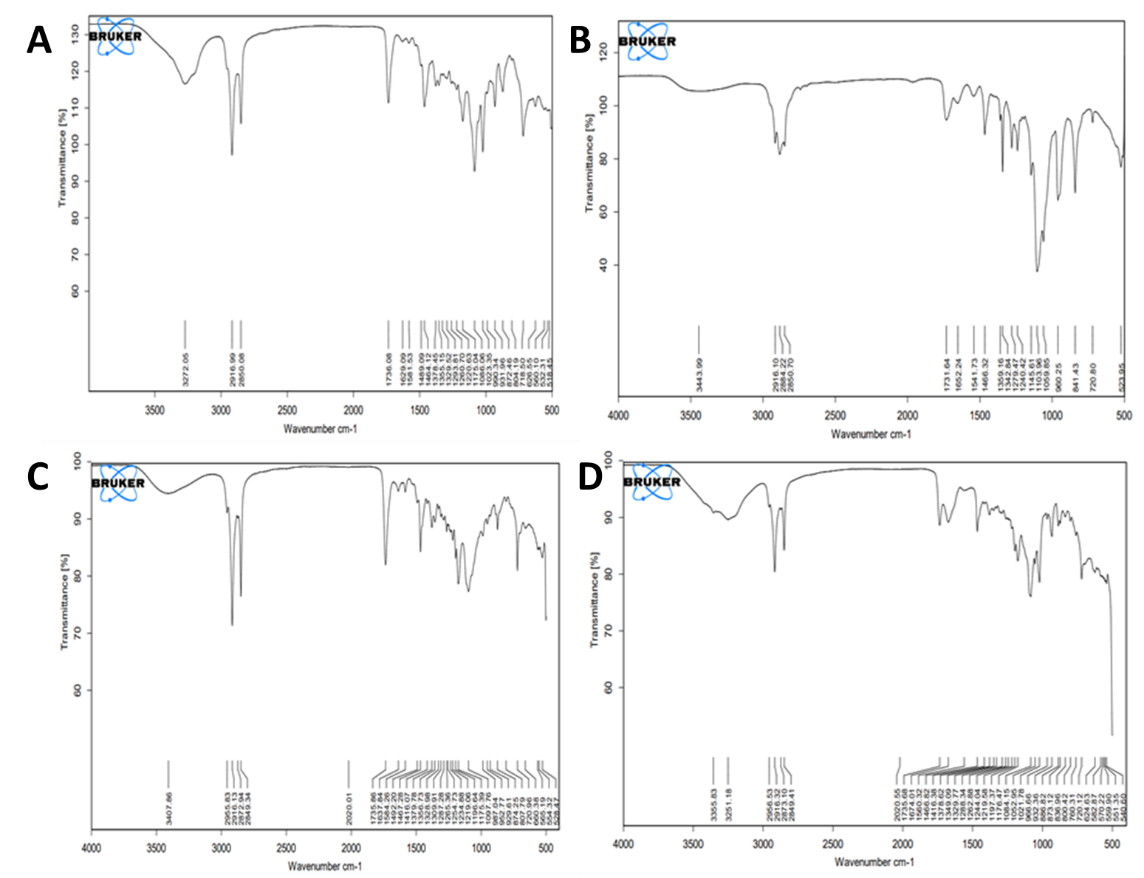


**Figure S6: FTIR spectra of (A) NVs-TM (B) DSPE-PEG-CCOH (C) NVs-COOH (D) NVs-CONH-PD**


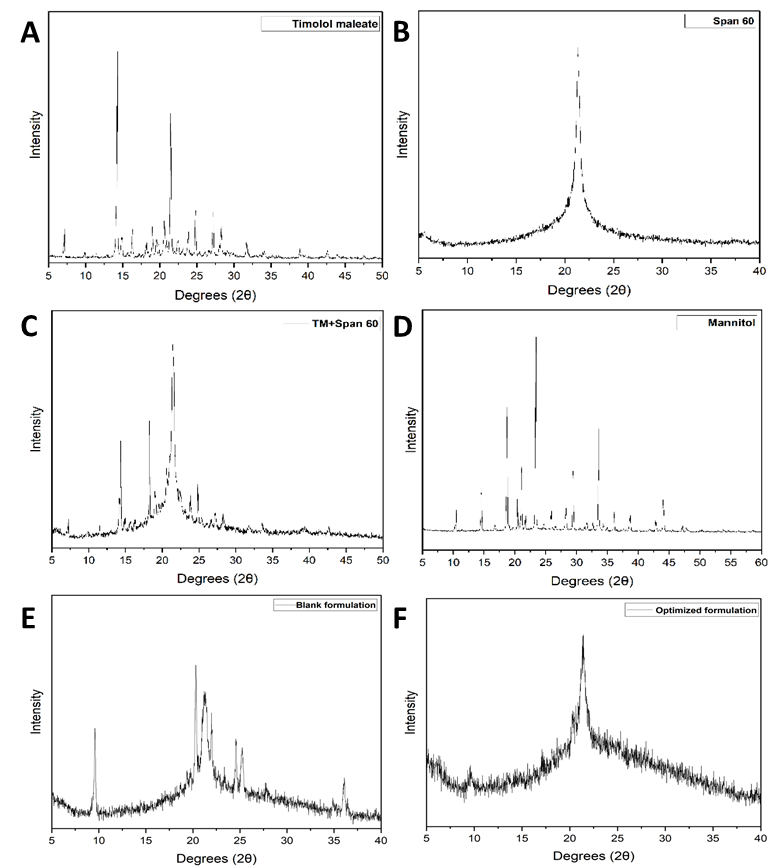


**Figure S7: XRD spectra of (A) TM (B)Span 60 (C)TM-Span 60 physical mixture (D)Mannitol (E) blank NVs (F) NVs-TM.**


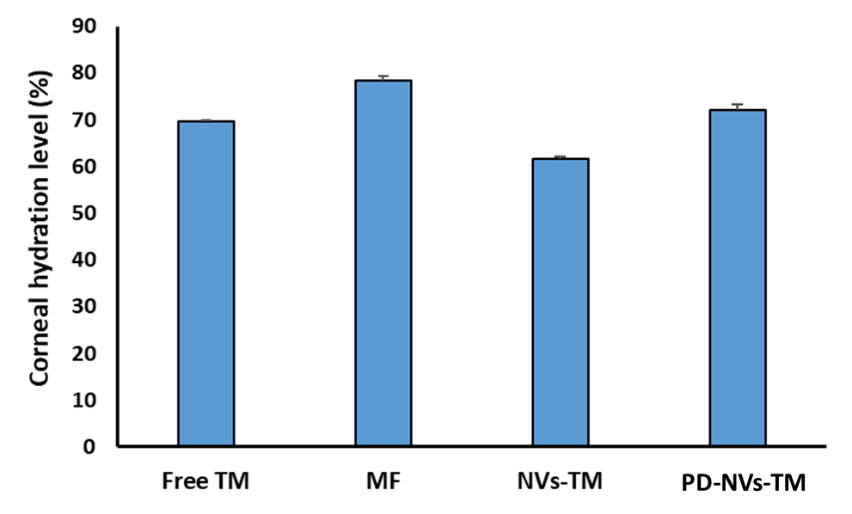


**Figure S8. Comparison of corneal hydration levels among the free TM solution, marketed formulation (MF), TM-loaded nanovesicles (NVs-TM), and peptide dendrimer-conjugated nanovesicles (PD-NVs-TM). Results are expressed as mean ± SD (n = 3).**

**
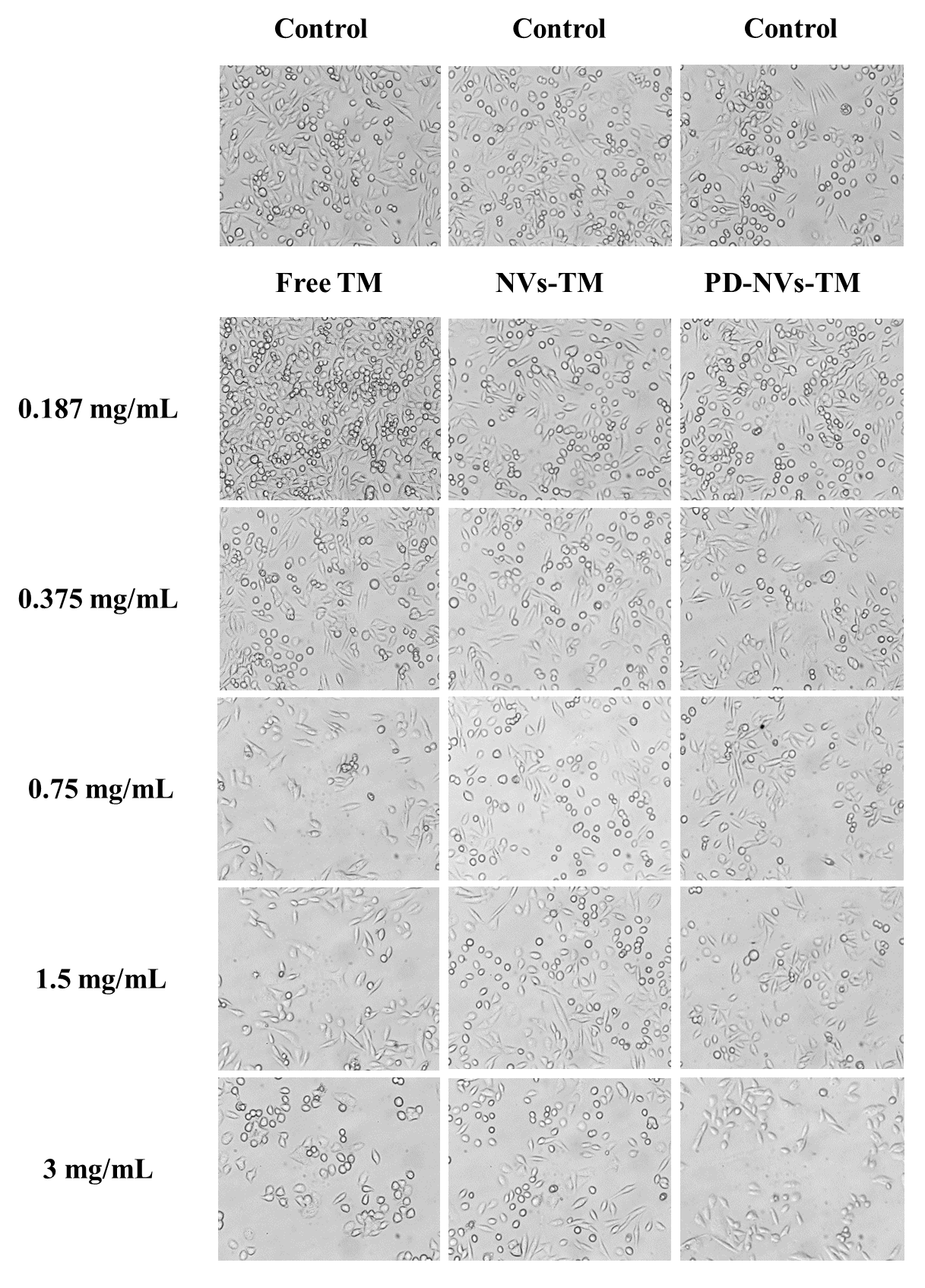
**

**Figure S9: Morphology of HCE-2 cells after treatment with various concentrations of free TM, NVs-TM and PD-NVs-TM**


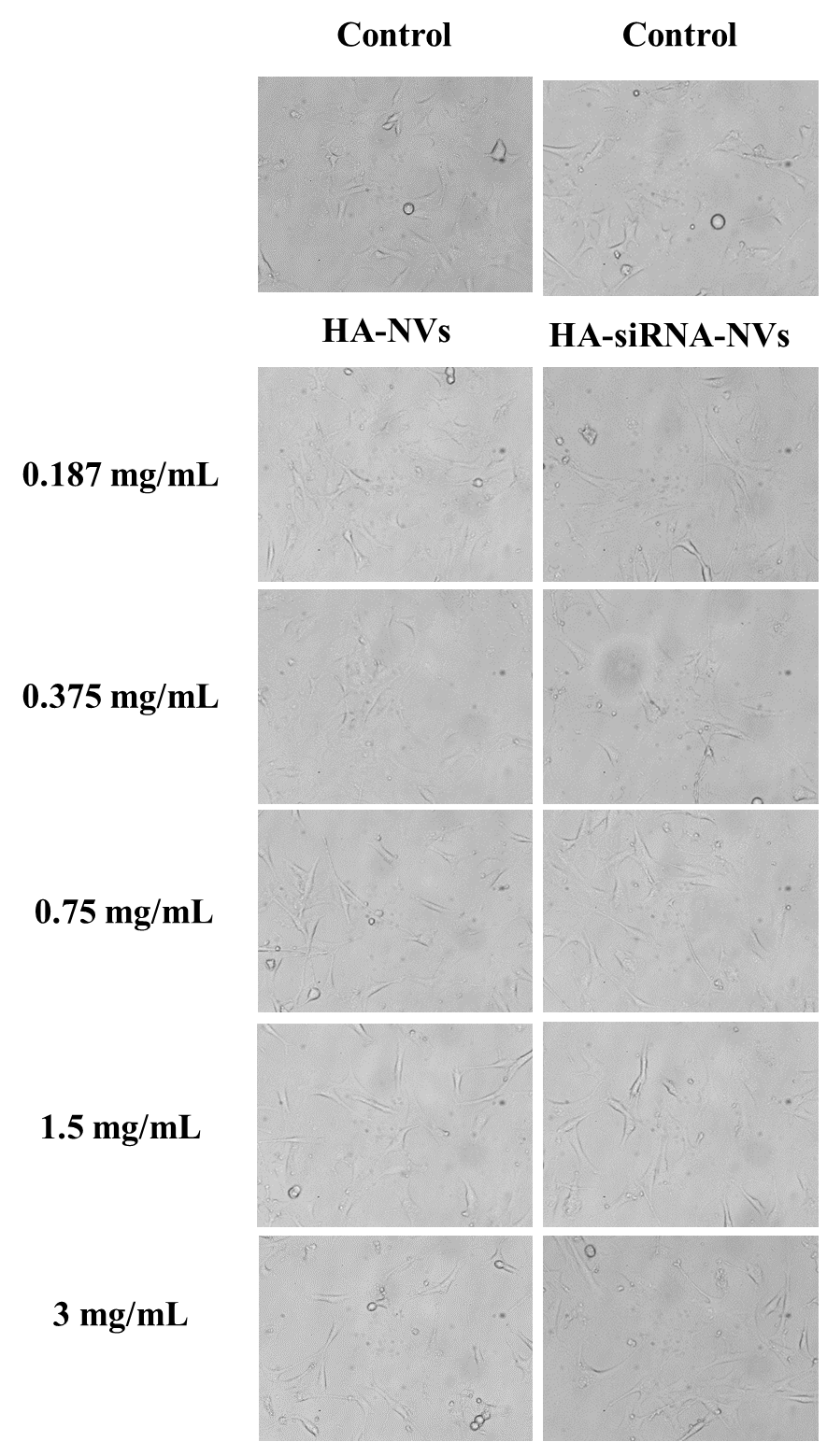


**Figure S10: Morphology of RGC-5 cells after treatment with various concentrations of HA-NVs and HA-siRNA-NVs.**

**Tables**

**Table S1. Box Behnken design for TM-loaded nano-vesicle formulation**

| **Factors (Independent variables)** | **Levels** | | |
| --- | --- | --- | --- |
|  | **Low (-1**) | **Middle (0**) | **High (+1**) |
| A: Span 60 concentration (mg) | 70 | 80 | 90 |
| B: Stirring speed (rpm) | 800 | 1000 | 1200 |
| C: Bath sonication time (mins) | 3 | 5 | 7 |
| **Responses (Dependent variables)** | **Required constraints** | | |
| R1: Particle size (nm) | Minimize | | |
| R2: Entrapment efficiency (%) | Maximize | | |

**Table S2. Experimental batches of nanovesicle formulation in Box-Behnken design**

| **Run** | **Span 60 conc. (mg)** | **Stirring speed (rpm)** | **Bath sonication time (min)** |
| --- | --- | --- | --- |
| 1 | 80 | 1200 | 3 |
| 2 | 80 | 800 | 3 |
| 3 | 90 | 1000 | 7 |
| 4 | 70 | 1000 | 3 |
| 5 | 80 | 1200 | 7 |
| 6 | 90 | 1000 | 3 |
| 7 | 80 | 800 | 7 |
| 8 | 70 | 1200 | 5 |
| 9 | 80 | 1000 | 5 |
| 10 | 90 | 1200 | 5 |
| 11 | 70 | 800 | 5 |
| 12 | 70 | 1000 | 7 |
| 13 | 80 | 1000 | 5 |
| 14 | 90 | 800 | 5 |
| 15 | 80 | 1000 | 5 |

**Table S3. Sense and Antisense strand sequence of caspase 2 gene silencing siRNA (GenBank: NM_007610.2)**

| **Number** | **Sequence position** | **Sense strand sequence** | **Antisense strand sequence** |
| --- | --- | --- | --- |
| siRNA-1 | 814-836 | CGACUUUUUGACAAUGCUA | UAGCAUUGUCAAAAAGUCG |
| siRNA-2 | 483-501 | GGAACACUCCUUAGAUAAU | AUUAUCUAAGGAGUGUUCC |
| siRNA-3 | 333-351 | GGGUCACUUGGAAGACUUA | UAAGUCUUCCAAGUGACCC |
| siRNA-4 | 870-888 | GCUUCAGCUCCAAGAGGUU | AACCUCUUGGAGCUGAAGC |
| siRNA-5 | 1208-1226 | UGGCCGACAUGCUUGUUAA | UUAACAAGCAUGUCGGCCA |
| siRNA-6 | 176-194 | ACCUCCUAGAGAAGGACAU | AUGUCCUUCUCUAGGAGGU |

**Table S4. The composition and the measured responses of the prepared TM-loaded nano-nano-vesicles formulae**

| **Run** | **A (mg)** | **B (rpm)** | **C (min)** | **R1 (nm)** | **R2 (%)** |
| --- | --- | --- | --- | --- | --- |
| 1 | 80 | 1200 | 3 | 208.7±6.4 | 96.03±8.99 |
| 2 | 80 | 800 | 3 | 414±3.4 | 89.11±2.31 |
| 3 | 90 | 1000 | 7 | 495.6±4.2 | 56.45±4.56 |
| 4 | 70 | 1000 | 3 | 182±6.5 | 42.32±6.11 |
| 5 | 80 | 1200 | 7 | 221.8±6.6 | 50.14±7.90 |
| 6 | 90 | 1000 | 3 | 482.3±5.4 | 69.83±8.23 |
| 7 | 80 | 800 | 7 | 225.7±2.1 | 51.5±6.45 |
| 8 | 70 | 1200 | 5 | 178±0.1 | 45.48±7.09 |
| 9 | 80 | 1000 | 5 | 390.1±8.3 | 59.25±2.66 |
| 10 | 90 | 1200 | 5 | 303.4±4.3 | 66.77±3.51 |
| 11 | 70 | 800 | 5 | 173.6±2.2 | 46.07±8.65 |
| 12 | 70 | 1000 | 7 | 214.1±1.6 | 36.19±6.78 |
| 13 | 80 | 1000 | 5 | 215.3±7.6 | 69.38±7.83 |
| 14 | 90 | 800 | 5 | 371.3±6.1 | 75.8±2.15 |
| 15 | 80 | 1000 | 5 | 211.4±4.7 | 78.95±4.70 |

A: Span 60 concentration; B: Stirring speed (rpm); C: Bath sonication time (mins);

R1: Particle size (nm); R2: Entrapment efficiency (%)

**Table S5. The constraints and the optimized formula suggested by DoE software**

| **Constraints** | **Goal** | **Lower** | **Upper** | **Lower** | **Upper** | **Importance** | |
| --- | --- | --- | --- | --- | --- | --- | --- |
| **Name** |  | **Limit** | **Limit** | **Weight** | **Weight** |  |  |
| **A: Span 60 conc. (mg)** | to be in range | 70 | 90 | 1 | 1 | 3 | |
| **B: Stirring speed (rpm)** | to be in range | 800 | 1200 | 1 | 1 | 3 | |
| **C: Bath sonication time (min)** | to be in range | 3 | 7 | 1 | 1 | 3 | |
| **R1: Particle size (nm)** | minimize | 173.60 | 495.6 | 1 | 1 | 3 | |
| **R2: EE (%)** | maximize | 60.00 | 96.03 | 1 | 1 | 3 | |
|  | | | | | | | |
| **Solutions** | **Span 60 conc. (mg)** | **Stirring speed (rpm)** | **Bath sonication time (min)** | **Particle size (nm)** | **EE (%)** | **Desirability** | |
| 1 | 70.30 | 1147.41 | 3.32 | 170.20 | 60.68 | 1 | Selected |

**Table S6. Model summary**

| **Responses** | **Particle size** | | **Entrapment efficiency** | |
| --- | --- | --- | --- | --- |
| F value | 6.126 | | 5.107 | |
| p-value | Model | 0.010498 | Model | 0.01868 |
|  | A | 0.001863 | A | 0.020355 |
|  | B | 0.045991 | B | 0.03011 |
|  | C | 0.571457 | C | 0.13382 |
| R^2^ value | 0.9994 | | 0.9928 | |
| Intercept | 285.82 | | 62.218 | |
| Equation terms with coefficient | 113.112 * A - 34.0875 * B - 16.225 * C | | 12.34875 * A -  0.5075 * B -  12.8763 * C | |
| Predicted value^a^ | 170.2±2.4 | | 60.65±3.1 | |
| Observed value^b^ | 178.9±7.3 | | 58.68±6.5 | |
| Percent relative error | -5.11 | | 3.24 | |

R^2^, coefficient of determination; terms are statistically significant if p<0.05; A, Span 60 concentration (mg); B, Stirring speed (rpm); C, Bath sonication time (mins).

^a^ Values predicted by Design-Expert® software for the optimized formulation.

^b^ Values obtained after taking batch (n = 3) for the optimized formulation

**Table S7. Physicochemical properties of plain and conjugated nanovesicles**

| **Formulations** | **PS (nm)** | **PDI** | **ZP (mV)** | **%EE of TM** |
| --- | --- | --- | --- | --- |
| blank nanovesicles  (NVs) | 178.9±4.212 | 0.202±0.018 | -35.5±0.442 | 58.68±3.120 |
| PD-conjugated nanovesicles  (PD-NVs-TM) | 282.3±3.961 | 0.305±0.002 | 22.6±0.871 | 53.21±4.863 |
| PEI coated nanovesicles  (PEI-NVs) | 303.4±2.721 | 0.438±0.014 | 32.5±0.331 | 56.11±4.243 |
| HA-coated siRNA loaded nanovesicles  (HA-siRNA-PEI-NVs) | 495.6±5.150 | 0.411±0.316 | -41.3±0.819 | 51.57±5.03 |

**Table S8. Cumulative drug release (µg) from Free TM, NVs-TM, and PD-NVs-TM formulations at different time intervals.**

| **Time (h)** | **Cumulative drug released (µg)** | | |
| --- | --- | --- | --- |
|  | **Free TM** | **NVs-TM** | **PD-NVs-TM** |
| 0.5 | 156 | 60.5 | 44.5 |
| 1 | 1280.5 | 130.5 | 67 |
| 2 | 1718 | 238.5 | 193 |
| 4 | 1986 | 765 | 678.5 |
| 6 | 2283 | 1271 | 921 |
| 8 | 2695.5 | 1544 | 1132.5 |
| 10 | 2995.5 | 1896 | 1486.5 |
| 12 | 3509.5 | 2061 | 1836 |
| 24 | 3827.5 | 2872.5 | 2456 |

**Table S9. *Ex vivo* corneal permeation of TM from free TM solution, marketed formulation, TM loaded nanovesicles and PD conjugated nanovesicles**

| **Time (h)** | **Percent amount permeated/cm2 (mean ± SD; n=3)** | | | |
| --- | --- | --- | --- | --- |
|  | **Free TM** | **MF** | **NVs-TM** | **PD-NVs-TM** |
| 0 | 0 | 0 | 0 | 0 |
| 0.5 | 2.165 ± 0.766 | 2.061 ± 0.719 | 2.889 ± 1.044 | 3.063 ± 0.757 |
| 1 | 7.453 ± 1.460 | 16.417 ± 4.892 | 15.705 ± 4.060 | 9.244 ± 0.127 |
| 2 | 13.057 ± 2.027 | 21.103 ± 0.688 | 24.403 ± 2.431 | 24.097 ± 0.277 |
| 3 | 20.403 ± 1.904 | 26.818 ± 2.808 | 38.833 ± 4.112 | 38.728 ± 0.264 |
| 4 | 25.002 ± 1.160 | 33.655 ± 2.040 | 44.112 ± 1.060 | 46.484 ± 0.111 |
| 6 | 28.779 ± 1.008 | 38.687 ± 1.949 | 50.434 ± 1.717 | 47.269 ± 0.198 |
| 8 | 37.389 ± 2.291 | 43.082 ± 1.542 | 53.177 ± 0.421 | 48.918 ± 0.051 |
| 10 | 42.957 ± 1.764 | 50.158 ± 1.818 | 53.972 ± 0.502 | 57.120 ± 0.033 |
| 12 | 47.870 ± 0.908 | 55.052 ± 0.318 | 60.429 ± 2.792 | 65.664 ± 0.140 |
| 24 | 65.164 ± 5.002 | 70.112 ± 5.252 | 83.589 ± 9.054 | 85.033 ± 0.369 |
| 48 | 81.347 ± 5.063 | 91.874 ± 5.700 | 96.516 ± 3.050 | 97.113 ± 0.111 |

**Table S10. Details of RNA concentration of different culture conditions quantified by Nanodrop**

| **Sample Name** | **Nucleic Acid (ng/µL)** | **A260/ A280** | **A260/ A230** |
| --- | --- | --- | --- |
| Control | 512.533 | 2.022 | 1.874 |
| Naked Positive siRNA | 277.318 | 1.931 | 1.665 |
| Optimised Positive siRNA | 572.615 | 1.938 | 1.563 |
| Optimised Negative siRNA | 131.369 | 1.809 | 1.026 |

**Table S11. Relative mRNA expression values of Caspase-2 in untreated and treated transfected RGC-5 cells by RT-PCR method**

| **Overlaid normalized gene expression of Test compounds in transfected RGC-5 cells (mean±SD; n=3)** | |
| --- | --- |
| **Culture condition/ Target gene** | **Caspase-2** |
| Control | 1.000±0.310 |
| Naked Positive siRNA | 0.180±0.098 |
| Opt Positive siRNA | 0.025±0.072 |
| Opt Negative siRNA | 1.250±0.102 |

**Table S12. Results of Acute eye irritation test**

| **Ocular tissues** | **Degree of ocular irritation** | | | | | | | |
| --- | --- | --- | --- | --- | --- | --- | --- | --- |
|  | **1 h** | | **24 h** | | **48 h** | | **72 h** | |
|  | **Control** | **Test** | **Control** | **Test** | **Control** | **Test** | **Control** | **Test** |
| **Group I (TM solution)** | | | | | | | | |
| Cornea | 0 | 0 | 0 | 0 | 0 | 0 | 0 | 0 |
| Iris | 0 | 0 | 0 | 0 | 0 | 0 | 0 | 0 |
| Conjunctiva | 0 | 0 | 0 | 2 | 0 | 1 | 0 | 1 |
| Chemosis | 0 | 0 | 0 | 0 | 0 | 0 | 0 | 0 |
| **Group II (NVs-TM)** | | | | | | | | |
| Cornea | 0 | 0 | 0 | 0 | 0 | 0 | 0 | 0 |
| Iris | 0 | 0 | 0 | 0 | 0 | 0 | 0 | 0 |
| Conjunctiva | 0 | 0 | 0 | 1 | 0 | 1 | 0 | 1 |
| Chemosis | 0 | 0 | 0 | 1 | 0 | 1 | 0 | 0 |
| **Group III (PD-NVs-TM)** | | | | | | | | |
| Cornea | 0 | 0 | 0 | 0 | 0 | 0 | 0 | 0 |
| Iris | 0 | 0 | 0 | 0 | 0 | 0 | 0 | 0 |
| Conjunctiva | 0 | 0 | 0 | 2 | 0 | 1 | 0 | 1 |
| Chemosis | 0 | 0 | 0 | 2 | 0 | 1 | 0 | 1 |

*Irritation was graded on a scale from 0 to 4 for cornea, 0 to 2 for iris, 0 to 3 for conjunctiva and 0 to 4 for Chemosis.

**Table S13. Results of Chronic eye irritation test**

| **Ocular tissues** | **Degree of ocular irritation** | | | | | | | |
| --- | --- | --- | --- | --- | --- | --- | --- | --- |
|  | **1 h** | | **24 h** | | **48 h** | | **72 h** | |
|  | **Control** | **Test** | **Control** | **Test** | **Control** | **Test** | **Control** | **Test** |
| **Group I (TM solution)** | | | | | | | | |
| Cornea | 0 | 0 | 0 | 0 | 0 | 0 | 0 | 0 |
| Iris | 0 | 0 | 0 | 0 | 0 | 0 | 0 | 0 |
| Conjunctiva | 0 | 0 | 0 | 1 | 0 | 0 | 0 | 0 |
| Chemosis | 0 | 0 | 0 | 0 | 0 | 0 | 0 | 0 |
| **Group II (NVs-TM)** | | | | | | | | |
| Cornea | 0 | 2 | 0 | 1 | 0 | 1 | 0 | 0 |
| Iris | 0 | 0 | 0 | 0 | 0 | 0 | 0 | 0 |
| Conjunctiva | 0 | 2 | 0 | 2 | 0 | 1 | 0 | 1 |
| Chemosis | 0 | 0 | 0 | 2 | 0 | 2 | 0 | 2 |
| **Group III (PD-NVs-TM)** | | | | | | | | |
| Cornea | 0 | 1 | 0 | 1 | 0 | 0 | 0 | 0 |
| Iris | 0 | 0 | 0 | 0 | 0 | 0 | 0 | 0 |
| Conjunctiva | 0 | 1 | 0 | 1 | 0 | 1 | 0 | 1 |
| Chemosis | 0 | 0 | 0 | 1 | 0 | 2 | 0 | 0 |

*Irritation was graded on a scale from 0 to 4 for cornea, 0 to 2 for iris, 0 to 3 for conjunctiva and 0 to 4 for Chemosis.

**Table S14. Mean IOP values in SD rats measured using iCare Home Tonometer after different treatments**

| **IOP readings (mm Hg**) **(mean ± SD; n=3**) | | | | | |
| --- | --- | --- | --- | --- | --- |
| Days | Group I Negative Control | Group II (Positive control) | Group III (MF) | Group IV (NVs-TM) | Group V  (PD-NVs-TM) |
| 0 | 13 ± 0.000 | 13 ± 2.081 | 13 ± 1.527 | 12 ± 0.577 | 13 ± 1.527 |
| 0* | 12 ± 0.471 | 26 ± 2.081 | 25 ± 1.527 | 23 ± 2.081 | 26 ± 1.527 |
| 0** | 13 ± 1.414 | 26 ± 1.732 | 23 ± 2.51 | 22 ± 1.527 | 23 ± 3.605 |
| 1 | 14 ± 0.000 | 24 ± 1.154 | 20 ± 1.732 | 19 ± 2.081 | 18 ± 2.516 |
| 2 | 13 ± 0.942 | 23 ± 2.081 | 18 ± 2.645 | 17 ± 2.081 | 15 ± 2.000 |
| 3 | 13 ± 0.882 | 23 ± 1.000 | 16 ± 4.041 | 16 ± 4.163 | 13 ± 0.577 |
| 4 | 12 ± 0.932 | 21 ± 1.000 | 15 ± 2.645 | 14 ± 2.516 | 11 ± 1.154 |
| 5 | 11 ± 0.000 | 21 ± 0.577 | 14 ± 2.645 | 13 ± 2.309 | 10 ± 1.000 |
| 6 | 11 ± 1.247 | 20 ± 1.154 | 11 ± 2.081 | 10 ± 1.154 | 8 ± 1.154 |
| 7 | 11 ± 0.942 | 18 ± 0.577 | 10 ± 1.527 | 9 ± 1.527 | 6 ± 1.154 |

0- Before glaucoma induction; 0*- 30 min after glaucoma induction; 0**- 30 min after treatment

**Table S15. Grading of histological changes seen in the cornea**

| **Group** | **Inflammatory infiltrate** | **Edema** | **Neovascularization** |
| --- | --- | --- | --- |
| Group I (1) | **-** | **-** | **-** |
| Group I (2) | **-** | **-** | **-** |
| Group I (3) | **-** | **-** | **-** |
| Group II (1) | **-** | **-** | **-** |
| Group II (2) | **-** | **-** | **-** |
| Group II (3) | **-** | **-** | **-** |
| Group III (1) | + | - | + |
| Group III (2) | **-** | **-** | **-** |
| Group III (3) | **-** | **-** | **-** |
| Group IV (1) | **-** | **-** | **-** |
| Group IV (2) | **-** | **-** | **-** |
| Group IV (3) | **-** | **-** | **-** |

**-** : Nil, + : Mild, ++ : Moderate
